# Magnetic order in Tsai-type icosahedral quasicrystals and periodic approximants

**DOI:** 10.1107/S2052252526006263

**Published:** 2026-09-01

**Authors:** Farid Labib, Ryuji Tamura

**Affiliations:** a Research Institute of Science and Technology, Tokyo University of Science, Tokyo 125-8585, Japan; b Department of Materials Science and Technology, Tokyo University of Science, Tokyo 125-8585, Japan; Tsinghua University, China

**Keywords:** quasicrystals, periodic approximants, magnetic order, Tsai-type compounds, inorganic materials, crystal design, aperiodic structures, materials science, magnetic structures, electron diffraction, transmission electron microscopy

## Abstract

Quasicrystals (QCs), a class of aperiodic crystals with long-range order and non-crystallographic rotational symmetry, provide a unique platform for exploring emergent magnetism beyond conventional periodic solids. Although theoretical studies have long suggested that magnetic order is compatible with quasi­periodicity, long-range magnetic order in real QCs has only recently been realized through design principles established from their periodic approximants. This review presents three complementary and hierarchical principles for understanding and controlling magnetism in Tsai-type QCs and periodic approximants: (i) tuning the valence electron concentration (*e*/*a*) to control the sign and strength of Ruderman–Kittel–Kasuya–Yosida (RKKY) interactions, (ii) exploiting crystal electric field (CEF) anisotropy to select spin orientation and stabilize noncoplanar magnetic textures, and (iii) controlling structural degrees of freedom to manipulate frustration. Together, these principles provide a phenomenological framework for understanding and organizing magnetic phase selection in quasiperiodic materials. They also provide a unified perspective on the contrasting emergence of long-range magnetic order and spin-glass-like freezing in Tsai-type quasicrystals. Critical behaviour near magnetic transitions, emerging opportunities and outstanding challenges are also discussed, highlighting future directions for discovering novel magnetic states in QCs.

## Abstract 2

This review summarizes recent experimental and theoretical advances in the magnetism of quasicrystals and their approximant crystals, and formulates three complementary and hierarchical design principles – valence electron concentration, crystal electric field-induced anisotropy and structural control of magnetic frustration – as primary tools for systematically tuning the magnetic ground states in these compounds.

## 1. Introduction

### 1.1. Overview of aperiodic crystals, quasicrystals and their periodic counterparts

According to the IUCr Dictionary of Crystallography (https://dictionary.iucr.org/Main_Page), a crystal is any mater­ial that produces a diffraction pattern in which the scattering intensity is concentrated in sharp Bragg peaks. Under this definition, crystals are broadly classified into periodic crystals, which possess three-dimensional (3D) translational symmetry, and aperiodic crystals, which exhibit long-range order despite lacking conventional 3D periodicity. Aperiodic crystals include incommensurately modulated crystals, incommensurate composite crystals and quasicrystals (QCs) (Janssen *et al.*, 2007 ▸). Their atomic structures cannot be described by a finite periodic unit cell in real space and are often represented using higher-dimensional crystallographic frameworks, where periodicity is recovered in superspace (van Smaalen, 2023 ▸). Therefore, the aperiodicity does not imply structural disorder, but rather a form of crystallinity beyond conventional periodic crystals.

**Figure 1:**
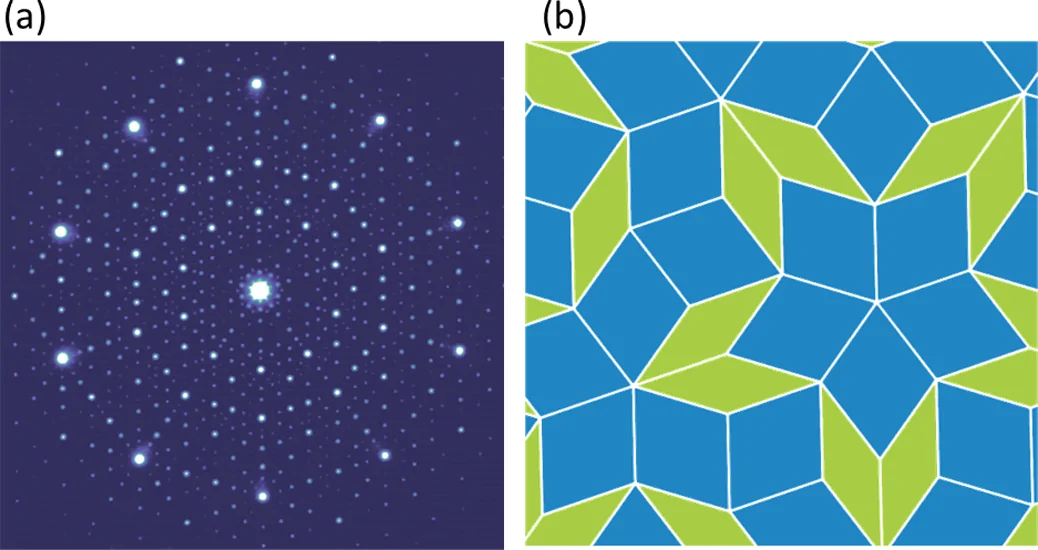
(*a*) Electron diffraction pattern of the Cd–Mg–Tm Tsai-type icosahedral quasicrystal (iQC), showing fivefold rotational symmetry. (*b*) An example of a section of a Penrose tiling comprised of two kinds of rhombi.

Among aperiodic crystals, QCs represent a particularly interesting class. The first experimental realization of a QC was reported by Shechtman in a metastable Al–Mn alloy in 1982 (published in 1984) (Shechtman *et al.*, 1984 ▸). QCs are well ordered non-periodic solids characterized by non-crystallographic rotational symmetries, such as eightfold, tenfold [Fig. 1 ▸(*a*)] and twelvefold symmetries, which are incompatible with 3D structure periodicity. In contrast to incommensurately modulated crystals, where main reflections and satellite reflections can be clearly distinguished, QCs possess a Fourier module in which all reciprocal basis vectors are equivalent, reflecting the absence of an underlying periodic lattice. Despite the absence of periodicity, their diffraction patterns consist of sharp Bragg reflections, demonstrating the existence of long-range order and crystallinity.

From a structural perspective, a minimal yet powerful representation of quasiperiodic structures is through aperiodic tilings, such as the Penrose tiling [Fig. 1 ▸(*b*)], in which the local motifs recur with well defined frequencies but in non-equivalent environments. Such tilings are not random. They exhibit long-range coherence without periodic repetition and can be rigorously constructed as projections of higher-dimensional periodic lattices (Grimm & Kramer, 2019 ▸). A representative example of a projected electron-density map of decagonal Al–Co–Ni superposed on the underlying Penrose tiling is provided elsewhere (Steurer, 2011 ▸).

**Figure 2:**
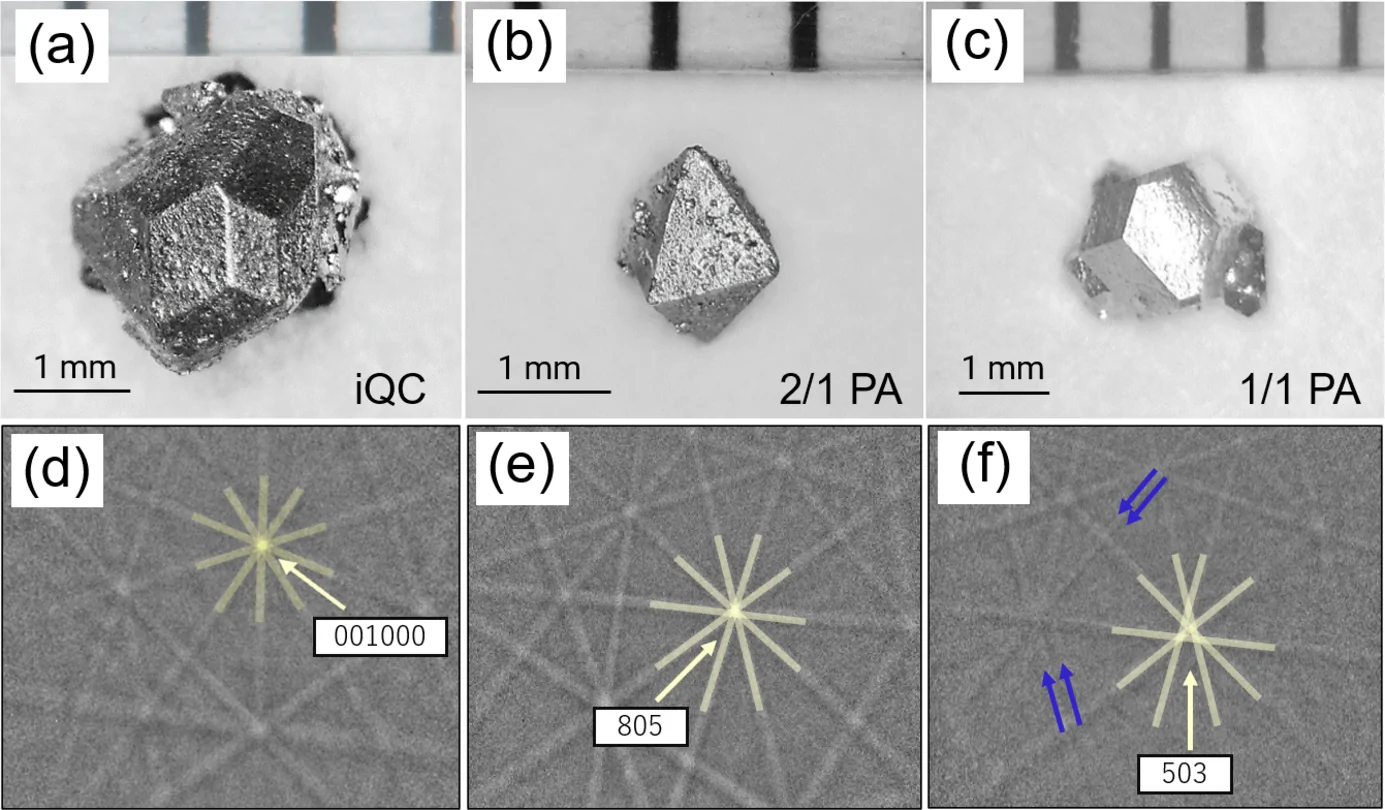
Optical microscopy images of single grains for the Cd–Mg–RE (*a*) iQC, (*b*) 2/1 PA and (*c*) 1/1 PA. The corresponding electron backscatter diffraction Kikuchi patterns showcasing the tenfold pole for the iQC and the pseudo-tenfold pole for the PAs are shown in panels (*d*)–(*f*). The images are adapted from Labib *et al.* (2020*a* ▸), copyright (2020) Elsevier, and Labib *et al.* (2019 ▸), copyright (2019) Taylor & Francis.

Closely related to QCs are their periodic counterparts, hereafter denoted as periodic approximants (PAs), which share essentially the same local atomic environment as their parent QCs but exhibit a periodic arrangement in physical space. Owing to their periodicity and the preservation of identical structural building units of QCs, PAs provide an ideal platform for disentangling physical properties that originate from the local cluster-scale environment from those governed by the global arrangement of clusters. It should be emphasized that the terms ‘quasicrystal’ and ‘approximant’ by no means imply inferior quality or crystallinity; both QCs and PAs are genuine crystals, characterized by long-range structural order and sharp Bragg diffraction. In fact, they are often thermally stable and can be grown as millimetre-sized single crystals. Figs. 2 ▸(*a*)–2(*f*) show representative QC, 2/1 PA and 1/1 PA single crystals in Cd–Mg–RE (RE = rare earth) systems belonging to Tsai-type variants (which will be discussed in Section 1.2.2) and their corresponding electron backscatter diffraction Kikuchi patterns showcasing tenfold (for the QC) and pseudo-tenfold (for the PAs) poles.

### 1.2. Structural details of quasicrystals

#### 1.2.1. Structural classification of icosahedral quasicrystals

Bragg reflections of icosahedral quasicrystals (iQCs) are described within a six-dimensional (6D) superspace formalism (Rokhsar *et al.*, 1987 ▸; Janssen, 1986 ▸). In this framework, symmetry analysis leads to superspace groups associated with three 6D cubic Bravais lattice types: primitive (P), face-centred (F) and body-centred (I). These types are distinguished by characteristic extinction conditions and scaling relations in reciprocal space. For example, F-type iQCs permit only all-even or all-odd reflection indices, whereas P-type iQCs impose no such restriction (Hahn, 2005 ▸). Although the 6D formalism allows P-, F- and I-type lattices, only P-type and F-type iQCs have been experimentally observed so far.

Based on their fundamental building motifs, iQCs are classified into three primary categories: (i) Al–transition metal systems (denoted as Mackay-type clusters), exemplified by Al–Cu–(Fe, Ru, Os) (Tsai *et al.*, 1987 ▸; Tsai *et al.*, 1988 ▸) and Al–Pd–(Mn, Re) (Tsai *et al.*, 1990 ▸; Fujita & Ogashiwa, 2021 ▸), (ii) Zn-based iQCs (Bergman-type clusters), such as Al–Li–Cu, Zn–Mg–Ga, Ti–Zr–Ni, Mg–Al–Pd and Zn–Mg–(Y, Dy, Gd, Ho, Tb, Er) (Niikura *et al.*, 1994 ▸; Ishimasa *et al.*, 2004 ▸), and (iii) Cd–RE iQCs (Tsai-type clusters) (Jarić & Gratias, 1989 ▸). Apart from Mackay-type iQCs, which are typically stabilized within a narrow electrons-per-atom (*e*/*a*) window around 1.75, Bergman- and Tsai-type iQCs exhibit a broader compositional flexibility with stable phases generally appearing in the range of *e*/*a* ≃ 2.0–2.15. The stricter *e*/*a* constraint in Mackay-type systems originates partly from the large valence mismatch among the constituent elements. For example, replacing one Fe atom by Al changes the electron count by approximately 5.66 electrons, resulting in limited solubility and rigid compositional stability.

In contrast, Bergman-type iQCs exhibit greater compositional tolerance. Since Zn and Mg are both divalent, their substitution causes little change in *e*/*a*, allowing partial chemical replacement. Once a favourable *e*/*a* is achieved, however, atomic size effects become dominant. In particular, the size-mismatch parameter δ = (*r*_*A*_ − *r*_*B*_)/*r*_*A*_, where *r*_*A*_ and *r*_*B*_ are the atomic radii of the constituent elements, limits Mg solubility to below ∼5 at.% in Bergman-type iQCs (Mizutani, 2011 ▸). Among the three classes, Tsai-type compounds exhibit the greatest chemical flexibility. By satisfying both *e*/*a* and δ conditions, the original binary Cd–RE systems have been extended into numerous ternary and multicomponent alloys, including Zn–*M*–RE (*M* = Mg, Mn, Fe, Co, Ni, Cu, Pd, Pt, Ag, Au), Cd–Mg–RE, Ag–Al–RE, Ag–In–RE, Al–Pd–RE, Al–Cu–RE, Ga–Cu–RE, Au–In–RE, Au–Ga–RE, Zn–Au–Yb/Tb, Au–Sn–Yb, Au–Ga–Yb and recently Ga–Pd–RE systems (Tsai, 2008 ▸; Steurer & Deloudi, 2008 ▸; Tsai, 2003 ▸; Tsai, 2013 ▸; Labib *et al.*, 2022*b* ▸).

#### 1.2.2. Structural framework of Tsai-type quasicrystals and periodic approximants

**Figure 3:**
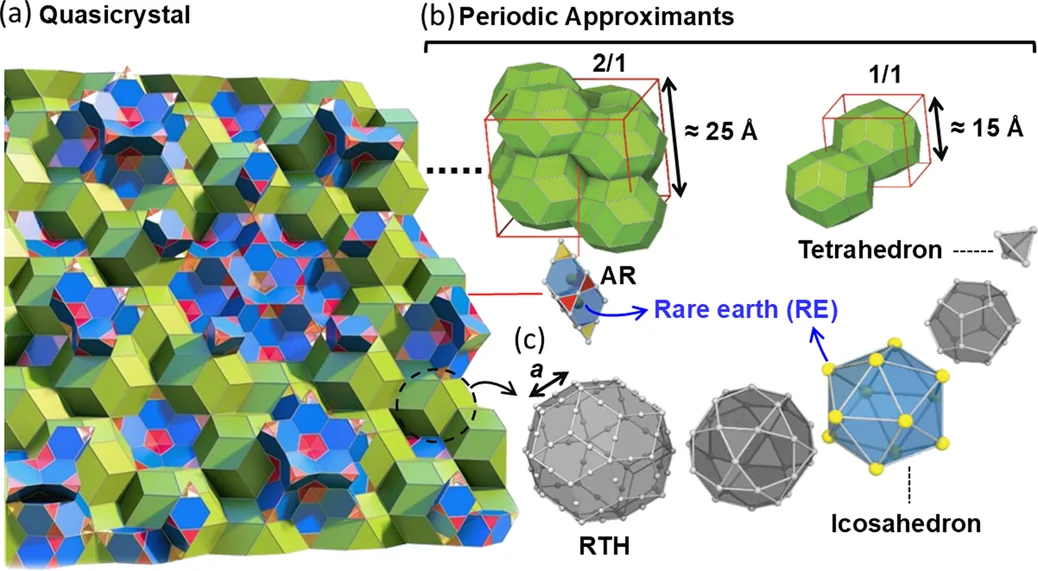
(*a)* A slab of i-YbCd_5.7_ in 3D physical space. (*b*) The packing of RTH units in the 2/1 and 1/1 cubic PAs. (*c*) The concentric shell structure of the RTH unit (from outermost to innermost): a Cd icosadodecahedron (30 atoms), an RE icosahedron (12 atoms), a Cd dodecahedron (20 atoms) and an inner Cd tetrahedron decorated by Cd atoms at the vertices and mid-edge positions. The RE atoms occupy two crystallographically distinct sites: the 12 vertices of the icosahedron and two sites located along the body-diagonal axis of the AR unit, which fills the interstitial space between neighbouring RTH units. The images are adapted from Takakura *et al.* (2007 ▸).

A representative illustration of the atomic configuration in the prototype Tsai-type Cd–Yb QC, 2/1 and 1/1 cubic PAs, is shown in Fig. 3 ▸. The space group of the QC was determined to be 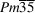. The Tsai-type 1/1 and 2/1 PAs (the two lowest orders of PAs within the higher-dimensional cut-and-projection framework) are cubic structures with lattice parameters of approximately *b* ≃ 15 Å and τ*b* ≃ 25 Å, respectively, where τ denotes the golden ratio (τ ≃ 1.618). In the rational cut-and-projection description, the lattice parameter *b* is given by 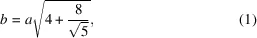
where *a* corresponds to the edge length of the surface rhombi of the rhombic triacontahedron (RTH) structural unit [Fig. 3 ▸(*c*)] and *b* represents the diameter of the RTH unit along its twofold axis. The 6D QC lattice parameter is related to *a* through 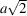 (Takakura *et al.*, 2007 ▸).

The 1/1 and 2/1 PAs differ in the manner in which the RTH units are packed and connected [Fig. 3 ▸(*b*)]. The 1/1 PA is characterized by a body-centred cubic packing of RTH units (space group 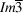), such that all atoms belong to the RTH framework, while the 2/1 PA (space group 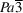) adopts a different packing scheme of RTH units, which requires the introduction of an additional building block to fill the interstitial gaps between neighbouring RTH units, that is, an acute rhombohedron (AR) with edge length *a* (Labib *et al.*, 2023 ▸; Yamada *et al.*, 2021 ▸; Gómez & Lidin, 2001 ▸). The AR contains two additional RE atoms along its long body-diagonal axis, while Cd atoms occupy the vertices and mid-edges [Fig. 3 ▸(*c*)]. For more information about the atomic structures of Tsai-type QCs and PAs, see Takakura *et al.* (2007 ▸).

A defining characteristic of Tsai-type compounds is the highly selective site occupation of the RE elements. In these structures, the RE atoms almost exclusively (and often fully) reside on the vertices of the icosahedral shell, while the remaining atomic sites are occupied by non-RE elements [Fig. 3 ▸(*c*)]. In higher-order PAs and QCs, additional RE atoms reside on specific sites along the long body-diagonal axis of the AR units. This pronounced chemical order plays a crucial role in the magnetic properties of Tsai-type compounds governed by the localized 4*f* electrons of the RE ions. Because the magnetic atoms occupy well defined crystallographic positions, the structural and magnetic degrees of freedom are largely decoupled from chemical disorder. As a result, the interpretation of experimental data becomes more straightforward, and theoretical modelling, such as crystal electric field (CEF) analysis or exchange interaction calculations, can be carried out with significantly reduced complexity compared with systems where magnetic ions are distributed over multiple inequivalent or partially occupied sites.

### 1.3. Overview of magnetism

#### 1.3.1. Magnetism in aperiodic crystals and quasicrystals

Magnetic order in aperiodic crystals has attracted long-standing interest due to the absence of conventional trans­lational symmetry, providing a fundamentally distinct platform for magnetic ordering compared with ordinary periodic solids. In such systems, complex magnetic states often emerge from the interplay of competing interactions under symmetry constraints. Notably, aperiodic magnetic order was recognized well before the modern structural definition of aperiodic crystals was established. Classic examples include incommensurately modulated antiferromagnetism in thulium (Koehler *et al.*, 1962 ▸; Brun *et al.*, 1970 ▸) and spin-density-wave (SDW) order in chromium (Fawcett, 1988 ▸), where magnetic satellite reflections appear at wavevectors incommensurate with the underlying atomic lattice.

Such behaviour is now widely observed in incommensurately modulated crystals, where magnetic order can develop either independent of, or in direct coupling with, an incommensurately modulated atomic lattice, leading to complex magnetic superspace structures (Perez-Mato *et al.*, 1999 ▸; Pato-Doldán *et al.*, 2023 ▸). In some cases, magnetic and structural modulations coexist or interact, resulting in highly intricate multi-*q* or higher-order magnetic states.

Complex incommensurate magnetic phases are also common in RE-containing frustrated magnets, where competing exchange interactions frequently stabilize SDW phases, helices, multi-*q* states and temperature-dependent incommensurate phases. Representative examples include Eu chalcogenides (Griessen *et al.*, 1971 ▸), Dy–Ge (Schobinger-Papamantellos *et al.*, 1992 ▸) and Tb–Ge (Schobinger-Papamantellos *et al.*, 1995 ▸), and pseudo-binary alloys such as REGe_1−*x*_Si_*x*_ (RE = Ce, Tb, Ho, Er) (Schobinger-Papamantellos *et al.*, 1994 ▸). In these materials, long-range oscillatory Ruderman–Kittel–Kasuya–Yosida (RKKY) interactions and geometric frustration generate multiple competing ordering vectors, often leading to multi-*q* or noncoplanar magnetic structures.

Here, it should be emphasized that the competing inter­actions often present in aperiodic systems do not necessarily suppress long-range magnetic order; rather, they can stabilize highly nontrivial ordered states and spatially complex and multi-scale magnetic moment structures. Recent studies have demonstrated that the interplay of competing exchange interactions, anisotropy and electronic structure can give rise to chiral magnetic order, vortex crystals and skyrmion lattices, often accompanied by topological transport phenomena such as the anomalous or topological Hall effect (Batista *et al.*, 2016 ▸; Witte *et al.*, 2026 ▸). In itinerant systems, field-tunable SDW phases further illustrate how delicate electronic instabilities can produce complex spatially modulated magnetic order, as exemplified in systems such as Sr_3_Ru_2_O_7_ (Lester *et al.*, 2021 ▸), where a magnetic field induces and controls SDW phases with distinct ordering wavevectors.

Within the broader context of aperiodic crystals, QCs represent a particularly intriguing class since quasiperiodicity is embedded directly into their atomic structure. Following the discovery of QCs, many experimental studies were devoted to understanding how the quasiperiodicity influences magnetic interactions and whether quasiperiodic lattices can host long-range magnetic order. In parallel with these experimental efforts, extensive theoretical studies investigated magnetism in QCs using a variety of models, as shall be briefly reviewed in Section 1.3.2. These studies demonstrated that quasi­periodicity itself does not fundamentally prohibit magnetic long-range order.

Another important aspect of QCs and PAs that has attracted long-standing interest is geometric frustration, particularly in Tsai-type systems. Geometric frustration arises when the topology of a magnetic structure prevents all magnetic interactions from being simultaneously satisfied (Mydosh, 1993 ▸). Such effects are often associated with structures composed of corner-sharing units, as found in kagome, triangular, Shastry–Sutherland and pyrochlore systems (Okamoto & Nomura, 1992 ▸; Shores *et al.*, 2005 ▸; Nakatsuji *et al.*, 2005 ▸; Sriram Shastry & Sutherland, 1981 ▸; Koga & Kawakami, 2000 ▸; Ramirez *et al.*, 1999 ▸; Harris *et al.*, 1997 ▸). This concept is particularly relevant to Tsai-type QCs and PAs, where RE ions form an interconnected network of corner-sharing octahedra (Labib *et al.*, 2022*b* ▸; Shiino *et al.*, 2025 ▸). The relatively high connectivity of this network may introduce competing magnetic interactions and provide a platform for unconventional magnetic states.

Despite these strong motivations, however, early experimental investigations of magnetic QCs consistently revealed spin-glass-like freezing rather than clear long-range magnetic order. Such behaviour was widely observed in Tsai-type systems, including Cd–RE (Goldman *et al.*, 2013 ▸), Cd–Mg–RE (Jagličić *et al.*, 2004 ▸; Sato *et al.*, 2002 ▸; Sebastian *et al.*, 2004 ▸; Labib *et al.*, 2020*a* ▸), Zn–Mg–RE (Kashimoto *et al.*, 2002 ▸; Sato *et al.*, 2006 ▸; Sato *et al.*, 2000 ▸; Fisher *et al.*, 2000 ▸; Dolinšek *et al.*, 2001 ▸) and Ag–In–RE (Ibuka *et al.*, 2011 ▸) iQCs. Similar glassy magnetic freezing was also reported in Mackay-type transition metal based iQCs (Hippert & Préjean, 2008 ▸; O’Handley *et al.*, 1990 ▸; Hauser *et al.*, 1986 ▸; Fukamichi *et al.*, 1986 ▸; Warren *et al.*, 1986 ▸). In Mackay-type iQCs, unlike their Tsai-type counterparts, localized magnetic moments are distributed more randomly throughout the quasiperiodic structure, resulting in magnetic behaviour analogous to that of dilute magnetic alloys. These experimental observations raised important questions regarding whether structural disorder and chemical randomness intrinsically suppress long-range magnetic order in real QCs, or whether genuinely ordered magnetic quasiperiodic states predicted by theoretical studies can ultimately be realized.

This long-standing issue was only recently resolved through the experimental discovery of long-range ferromagnetic (FM) (Tamura *et al.*, 2021 ▸; Takeuchi *et al.*, 2023 ▸) and antiferromagnetic (AFM) (Tamura *et al.*, 2025 ▸) orders in real iQCs. In particular, the discovery of an AFM iQC represents a major breakthrough in condensed-matter physics, several decades after theoretical studies had already established that quasiperiodicity itself does not fundamentally prohibit AFM long-range order. These discoveries have shifted the central focus of the field from the fundamental question of *whether long-range magnetic order can exist in real QCs* to the more advanced challenge of *how magnetic order in quasiperiodic systems can be systematically controlled and ultimately designed.*

#### 1.3.2. Theoretical background on magnetism of quasicrystals

One of the earliest theoretical studies on magnetism in quasiperiodic systems was performed by Godrèche and co-workers, who investigated an Ising model on the Penrose tiling using a Migdal–Kadanoff real-space renormalization approach (Godrèche *et al.*, 1986 ▸). Their work showed that quasiperiodicity can generate frustration without randomness and introduced the concept of ‘quasiferromagnetic’ order, providing early evidence that quasiperiodicity can fundamentally modify magnetic phase behaviour.

Subsequent studies addressed the microscopic origin of magnetic interactions. Using a tight-binding model on the octagonal tiling, Jagannathan (1994 ▸) demonstrated that magnetic susceptibility and local electronic properties depend strongly on the coordination environment, while RKKY-like interactions remain possible even without translational symmetry. Roche and Mayou further developed a real-space formalism for RKKY interactions in aperiodic systems and showed that quasiperiodic electronic states strongly affect the amplitude and sign of indirect exchange interactions (Roche & Mayou, 1999 ▸). Around the same time, Lifshitz established a higher-dimensional symmetry framework for magnetic neutron diffraction in QCs and showed that AFM order is symmetry-allowed in P- and I-type iQCs but forbidden in F-type systems (Lifshitz, 1998 ▸; Lifshitz, 2000 ▸).

Further insight into quasiperiodicity-induced frustration was provided by Vedmedenko *et al.* (2004 ▸), who studied classical spins on octagonal tilings with competing interactions. Their results showed that quasiperiodic geometry alone can stabilize noncollinear magnetic states and produce magnetic subtilings with distinct local frustration parameters.

Quantum Monte Carlo, spin-wave and renormalization-group studies of AFM Heisenberg models on Penrose and octagonal tilings further demonstrated that long-range Néel order can survive in quasiperiodic systems despite strong site-dependent variations of ordered moments (Wessel *et al.*, 2003 ▸; Jagannathan, 2004 ▸; Jagannathan *et al.*, 2007 ▸). In particular, Jagannathan and co-workers showed that the Penrose AFM exhibits a hierarchical distribution of local staggered magnetization associated with quasiperiodic local environments (Jagannathan *et al.*, 2007 ▸).

The role of quasiperiodicity in critical phenomena was examined by Ledue and co-workers, who showed that the FM Ising model on the octagonal tiling belongs to the conventional 2D Ising universality class (Ledue *et al.*, 1995 ▸). Subsequent studies by Thiem and Chalker reported spatially inhomogeneous correlations and unconventional magnetic responses in quasiperiodic Ising systems with competing interactions (Thiem & Chalker, 2015*a* ▸; Thiem & Chalker, 2015*b* ▸).

More recently, theoretical attention has shifted toward Tsai-type magnetic QCs. In Tb-based iQCs, CEF-induced anisotropy on Tb_12_ icosahedra was shown to stabilize noncoplanar textures including hedgehog, antihedgehog, whirling and antiwhirling states (Watanabe, 2021*b* ▸; Watanabe, 2021*a* ▸).

### 1.4. Scope and strategy of this review

Based on experimental observations and theoretical modelling, this review is intended as a practical guide for the design and realization of magnetic orders in QCs and PAs. At the same time, it provides a unified framework for understanding the contrasting emergence of long-range magnetic order and spin-glass-like freezing in Tsai-type QCs. This review introduces three complementary and hierarchical design principles for controlling magnetism in Tsai-type QCs and PAs. The first is tuning the valence electron concentration to control the sign and strength of RKKY-mediated exchange interactions. The second is controlling spin degrees of freedom through CEF-induced single-ion anisotropy, which constrains magnetic moment orientation and stabilizes nontrivial magnetic textures. The third is to exploit various structural degrees of freedom inherent in Tsai-type structures to manipulate electronic and magnetic states.

Beyond magnetic ground states, this review also examines the critical behaviour near the magnetic phase transition in QCs and PAs. Finally, emerging opportunities and current challenges are discussed, highlighting future directions for the rational design of magnetic states in quasiperiodic materials.

Here, it should be emphasized that the present review focuses on Tsai-type QCs and PAs and does not cover the Mackay- or Bergman-type families. This focus is for two main reasons. First, as discussed earlier, Tsai-type iQCs exhibit exceptionally strong site selectivity for RE elements [Fig. 3 ▸(*c*)], which simplifies the investigation of magnetism arising from localized 4*f* moments governed by CEF effects and RKKY-mediated exchange interactions. The situation, however, differs markedly in, for example, Mackay-type iQCs, where localized magnetic moments are more randomly distributed throughout the quasiperiodic structure, often leading to glassy magnetic freezing similar to that observed in dilute magnetic alloys.

The second reason is the remarkable compositional flexibility of the Tsai-type family, which has enabled the development of a wide variety of ternary and multicomponent alloys, significantly expanding the number of experimentally accessible compounds. As a result, sufficient experimental data have been accumulated to reveal systematic trends and establish empirical guidelines for the emergence of magnetic order, which form the central theme of this review. This focus, however, does not imply that Mackay- or Bergman-type iQCs are of lesser importance in terms of their magnetism. On the contrary, these families represent highly promising platforms for future studies of emergent magnetism because of their distinct structural and electronic characteristics compared with Tsai-type systems. For instance, the inherently low *e*/*a* values of Mackay-type iQCs already provide an intriguing playground for exploring emergent magnetic ground states and correlation effects in quasiperiodic structures that have not been seen yet.

## 2. Design principle I: valence electron concentration (*e*/*a*)

### 2.1. Universal *e*/*a* dependence of exchange interactions in periodic approximants

The valence electron concentration, commonly expressed as the electron-per-atom (*e*/*a*) ratio, has long served as a unifying descriptor for electronic structure and phase stability in intermetallic compounds. A wide variety of well known crystal structures preferentially stabilize within characteristic *e*/*a* values: body-centred cubic structures near *e*/*a* ≃ 1.5, complex cubic phases including γ-brass around *e*/*a* ≃ 1.62, hexagonal close-packed phases near *e*/*a* ≃ 1.75 and Tsai-type and Bergman-type QCs and PAs typically close to *e*/*a* ≃ 2.0 (Ferro & Saccone, 2008 ▸; Fujiwara & Ishii, 2008 ▸). This systematic correspondence highlights the central role of electron concentration in governing both structural stability and electronic states in intermetallic compounds.

The influence of *e*/*a* on magnetic properties has traditionally been discussed within the Slater–Pauling framework, which correlates magnetic moments with valence electron count (Kübler, 2009 ▸). Recent studies of a variety of non-stoichiometric Tsai-type Au–SM–RE (SM = semimetal) 1/1 PAs have shown exceptional flexibility in Au/SM substitution beyond 33 at.% without breaking the underlying crystallographic symmetry (Labib *et al.*, 2026*b* ▸). Note that, in non-stoichiometric compounds, compositional flexibility allows the valence electron concentration to be adjusted without altering the fundamental crystal symmetry. Given the distinct electron valence numbers of Au and SM elements, such substitution allows a systematic reduction of *e*/*a* from an original value of approximately 2.0 down to 1.6. Such a reduction in *e*/*a* has been shown to induce a sequence of ordered FM and AFM ground states (Ishikawa *et al.*, 2018 ▸; Labib *et al.*, 2025 ▸; Labib *et al.*, 2022*a* ▸; Labib *et al.*, 2026*b* ▸).

### 2.2. *e/a*-guided realization of ferromagnetic and antiferromagnetic orders in periodic approximants

**Figure 4:**
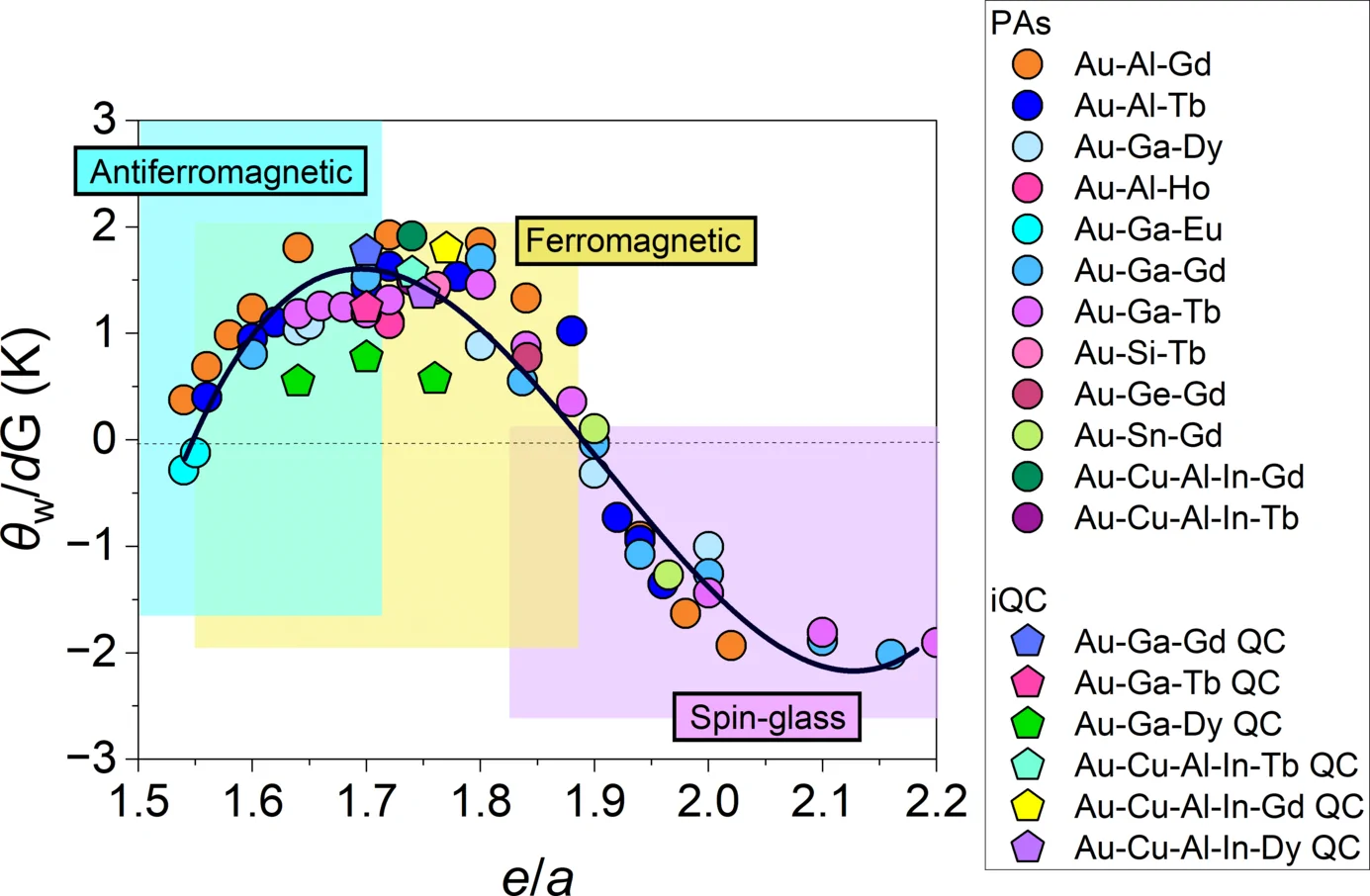
The electron-per-atom (*e*/*a*) dependence of θ_W_/*d*G for PAs exhibiting different magnetic ground states. Solid curves represent polynomial fits to the θ_W_/*d*G data. The background shading highlights regions corresponding to antiferromagnetic (AFM), ferromagnetic (FM) and spin-glass phases. The θ_W_/*d*G values of the newly discovered FM QCs are also plotted, as pentagon markers, and they fall within the FM region of the universal θ_W_/*d*G–*e*/*a* phase diagram.

Fig. 4 ▸ presents the evolution of the paramagnetic Curie–Weiss temperature θ_W_ as a function of *e*/*a* across a broad range of magnetic 1/1 PAs. To enable direct comparison among different RE systems, the values of θ_W_ are normalized by the de Gennes factor, *d*G = (*g*_*J*_ − 1)^2^*J*(*J* + 1), where *g*_*J*_ and *J* are the Landé *g* factor and the total angular momentum of the RE ion, respectively. Since θ_W_ provides a measure of the net magnetic exchange interaction, positive (respectively, negative) values correspond to predominantly FM (respectively, AFM) coupling. In Fig. 4 ▸, the *e*/*a* values are evaluated using Mizutani’s valence electron scheme (Mizutani, 2011 ▸). Interestingly, despite the wide chemical diversity of the alloys studied and variations in the RE elements, θ_W_ follows a universal oscillatory dependence on *e*/*a*. At higher *e*/*a* values (above ∼1.83), θ_W_ is predominantly negative, indicating dominant AFM interactions; it then evolves into a strongly positive regime around *e*/*a* ≃ 1.75, before gradually decreasing toward weakly positive values at lower *e*/*a*.

The universal oscillatory dependence of θ_W_ on *e*/*a* observed in PAs provides simple but strong experimental evidence that magnetic exchange interactions in these compounds are governed by the conduction-electron-mediated RKKY mechanism. Within this framework, the oscillatory character of the exchange interaction arises from the Friedel term in the interaction potential, expressed as 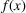 = 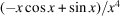, where *x* = 2*k*_F_*r*, *k*_F_ is the Fermi wavevector and *r* denotes the distance between two localized spins in physical space. Under the free-electron approximation, the Fermi wavevector scales as *k*_F_ = (3π^2^*N*/*V*)^1/3^, with *N*/*V* representing the conduction-electron density, which is controlled in practice by the valence electron concentration *e*/*a*. Therefore, tuning *e*/*a* provides a direct means of controlling both the magnitude and the sign of the magnetic exchange interactions.

The systematic variation of the Weiss temperature θ_W_ shown in Fig. 4 ▸ reveals a robust design strategy for realizing magnetic order in PAs through controlled tuning of the valence electron concentration. In particular, an *e*/*a* value around 1.7, where θ_W_ reaches its maximum positive values, emerges as a key value for stabilizing FM order in Tsai-type systems. Lately, as shall be discussed in Section 2.4, it has been revealed that such an *e*/*a* criterion, originally established based on PA datasets, remains broadly applicable to QCs as well. Following such a simple *e*/*a*-based guideline, a new family of FM iQCs with *e*/*a* close to 1.7 was recently designed and synthesized via melt-spinning of arc-melted alloys with nominal compositions Au_65_Ga_20_RE_15_ (RE = Gd, Tb and Dy) (Tamura *et al.*, 2021 ▸; Takeuchi *et al.*, 2023 ▸). More recently, bulk FM QCs with high structural coherence and thermal stability were discovered in quaternary Au–Cu–Al–In–RE (RE = Gd, Tb, Dy) iQCs (Tamura *et al.*, 2026 ▸). These materials were identified as P-type iQCs exhibiting long-range FM order, with Curie temperatures ranging from *T*_C_ = 7.5 K to 28.3 K depending on the RE variant. Notably, the corresponding θ_W_ values (pentagon symbols in Fig. 4 ▸) closely match those of their PA counterparts (filled circles in Fig. 4 ▸) in both sign and magnitude, which indicates that RKKY-mediated exchange interactions are largely preserved in QCs despite the absence of translational periodicity, provided that the *e*/*a* values are comparable.

Overall, Fig. 4 ▸ may be regarded as a phenomenological magnetic phase diagram of Tsai-type compounds, summarizing a large body of experimental observations across different alloy families, RE elements and structural variants. Although it does not uniquely determine the magnetic ground state in every compound (as will be discussed in the following section), it provides a useful organizing framework for understanding magnetic phase selection in Tsai-type QCs and PAs.

### 2.3. Limitations of the *e/a* scale

Despite the above universality in the strength and magnitude of magnetic exchange interactions, *e*/*a* fails to provide a unified framework for the underlying magnetic ground states from several aspects. One clear limitation is that the phase boundaries shown in Fig. 4 ▸ are not sharply defined, as reflected by the overlap of the background colours representing the magnetic ground states. Instead, the locations of these boundaries systematically depend on the choice of RE or SM elements in, for example, Au–SM–RE alloy systems. This dependence is nontrivial, as compounds sharing nearly identical nominal compositions but containing different RE ions can stabilize fundamentally distinct magnetic ground states. For example, Au_65_Ga_21_Tb_14_ (Nawa *et al.*, 2023*a* ▸) exhibits AFM order, whereas Au_65_Ga_21_Dy_14_ (Labib *et al.*, 2026*a* ▸) displays FM order. A comparable shift in *e*/*a* values corresponding to the magnetic ground-state boundaries is also observed upon substitution of the SM element within the Au–SM–RE family, such as replacing Ga with Al. These results indicate the limitations of the rigid band approximation in the *e*/*a* scaling scheme in classifying magnetic ground states in QCs and PAs of different alloy families within a single universal picture. This inconsistency stems from the fact that subtle modifications of the electronic band structure induced by the change in the RE ion or SM species can alter the Fermi surface topology and, consequently, the magnetic exchange interactions, even though the total number of valence electrons may remain unchanged.

Within the *e*/*a*-based classification, QCs and PAs located in the region labelled ‘spin-glass’ in Fig. 4 ▸ typically display frozen spin-glass-like states. This trend, however, is not universal either, as several prominent exceptions exhibiting long-range magnetic order have been reported within the spin-glass region. Representative examples include the AFM Ga_50_Pd_36_Tb_14_ 2/1 PA (Labib *et al.*, 2022*b* ▸; So *et al.*, 2020 ▸) and the AFM Cd_6_RE (RE = Gd, Tb, Dy, Ho, Er, Tm) 1/1 PAs (Mori *et al.*, 2012 ▸; Tamura *et al.*, 2010 ▸). These observations further highlight the limitations of the simple *e*/*a*-based class­ification in fully describing the magnetic ground states and microscopic magnetic properties of Tsai-type QCs and PAs. In this sense, Fig. 4 ▸ should be regarded primarily as a phenomenological organizing framework that captures the major experimental tendencies of magnetic phase selection.

### 2.4. Case study 1: discovery of ferromagnetic quasicrystals

As discussed earlier, one of the central conclusions drawn from Fig. 4 ▸ is that long-range magnetic order emerges in Tsai-type PAs upon reducing the average *e*/*a* across a wide range of alloy systems, RE elements and PA orders (*e.g.* 1/1 or 2/1). This establishes *e*/*a* tuning as a promising strategy for designing QCs with long-range magnetic order. A major technical challenge, however, has been how to stabilize iQCs with *e*/*a* ≃ 1.7 or lower, given that Tsai-type iQCs are generally stabilized in a range of *e*/*a* ≃ 2.00–2.15 in the original form. By combining multiple materials-processing approaches, Au_65_Ga_20_RE_15_ (RE = Gd, Tb and Dy) iQCs with *e*/*a* close to 1.70 were recently synthesized (Tamura *et al.*, 2021 ▸; Takeuchi *et al.*, 2023 ▸) via rapid quenching of the melt and confirmed to exhibit FM order, demonstrating that the *e*/*a* tuning strategy is indeed applicable to quasiperiodic systems.

**Figure 5:**
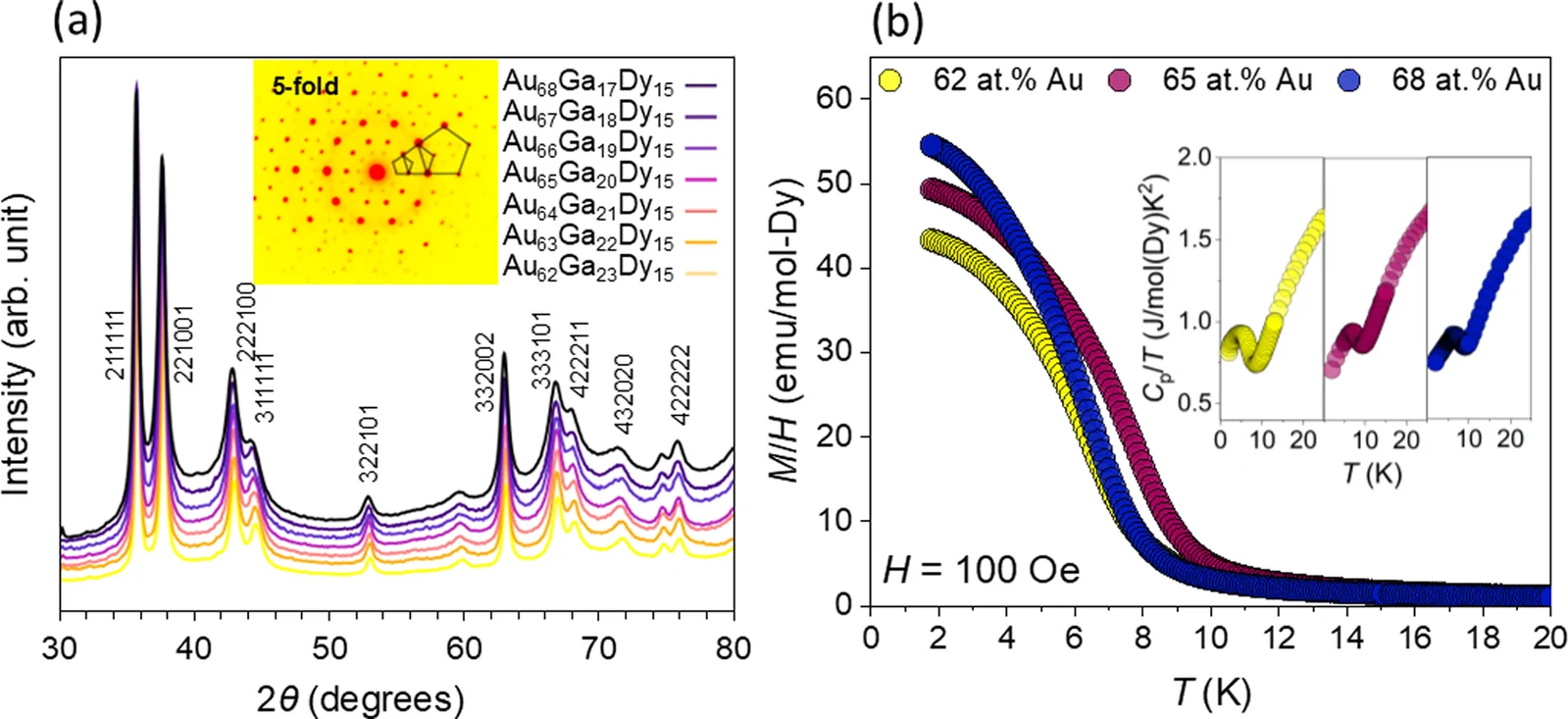
(*a*) Powder X-ray diffraction patterns of the Au_*x*_Ga_85−*x*_Dy_15_ (*x* = 62–68) iQCs. In all patterns, the peaks can be indexed as those of a primitive icosahedral QC, indicating the formation of highly pure single-phase QCs. The inset shows a selected-area electron diffraction pattern of the Au_65_Ga_20_Dy_15_ QC taken along the fivefold axis. (*b*) The temperature dependence of the field-cooled magnetic susceptibility (*M*/*H*) for the Au_*x*_Ga_85−*x*_Dy_15_ (*x* = 62, 65 and 68) iQCs. The inset in (*b*) shows the temperature dependence of *C*_p_/*T* for the same samples in the range 0 ≤ *T* ≤ 25 K.

In the case of RE = Dy (Takeuchi *et al.*, 2023 ▸), for example, a single-phase FM iQC region was found to extend over a relatively wide compositional range, Au_65±*x*_Ga_20±*x*_Dy_15_ (*x* = 3), marking the first compositionally tunable FM QC reported to date [see Fig. 5 ▸(*a*) for the powder XRD patterns]. The Curie temperature of the Au_65_Ga_20_Dy_15_ QC, for example, is *T*_C_ ≃ 7.5 K [Fig. 5 ▸(*b*)]. The *C*_p_/*T* curves [inset of Fig. 5 ▸(*b*)] clearly exhibit sharp anomalies corresponding to the divergence of susceptibility in *M*/*H* at *T*_C_ [Fig. 5 ▸(*b*)], confirming the occurrence of an FM transition. In the field-dependent magnetization (*M*–*H*) curve measured at 1.8 K, the magnetization is limited to about 6.9 μ_B_/Dy^3+^, which corresponds to roughly two-thirds of the full free-ion moment of Dy^3+^ (*g*_*J*_μ_B_ = 10μ_B_/Dy^3+^).

**Figure 6:**
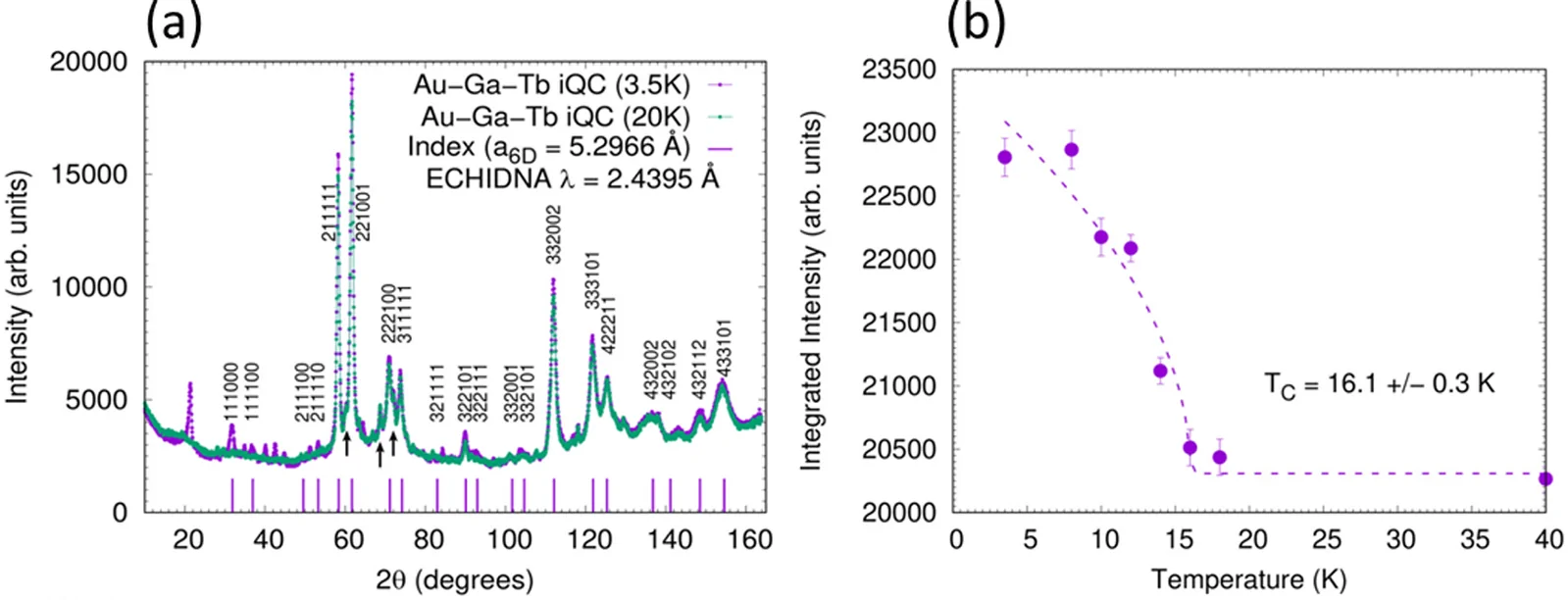
(*a*) Powder neutron diffraction patterns of the Au_65_Ga_20_Tb_15_ iQC measured at the base temperature (3.5 K) and at a paramagnetic temperature (20 K), *i.e.* below and above *T*_C_ = 16 K inferred from bulk measurements. Calculated nuclear peak positions and their 6D indices are also shown. The calculations were performed using a 6D lattice parameter of *a*_6D_ = 5.2966 Å. Vertical arrows indicate nuclear Bragg reflections originating from a minor impurity phase corresponding to the Au–Ga–Tb 1/1 PA. The magnetic [111000] peak is clearly observed at 2θ = 31.8° below *T*_C_ and disappears above *T*_C_, providing direct evidence for the formation of long-range magnetic order in the Au_65_Ga_20_Tb_15_ iQC. (*b*) The temperature dependence of the integrated intensity of the [111000] reflection. A fit to the empirical relation *I* ∝ (1 − *T*/*T*_C_)^2β^ yields an estimated transition temperature of *T*_C_ = 16.1 (3) K.

In the case of the Au_65_Ga_20_Tb_15_ iQC (Tamura *et al.*, 2021 ▸), neutron diffraction measurements have provided further microscopic evidence for FM order establishment. In this iQC, distinct magnetic Bragg reflections appear below *T*_C_ = 16 K, with the two strongest peaks indexed as [111000] and [111100] [Fig. 6 ▸(*a*)]. The temperature dependence of the [111000] magnetic Bragg reflection intensity [Fig. 6 ▸(*b*)] shows a clear rise below *T*_C_ = 16.1 (3) K, which is in excellent agreement with the value of *T*_C_ = 16 K obtained from bulk magnetic measurements. These observations provided solid evidence for the formation of FM order in the Au_65_Ga_20_Tb_15_ iQC.

**Figure 7:**
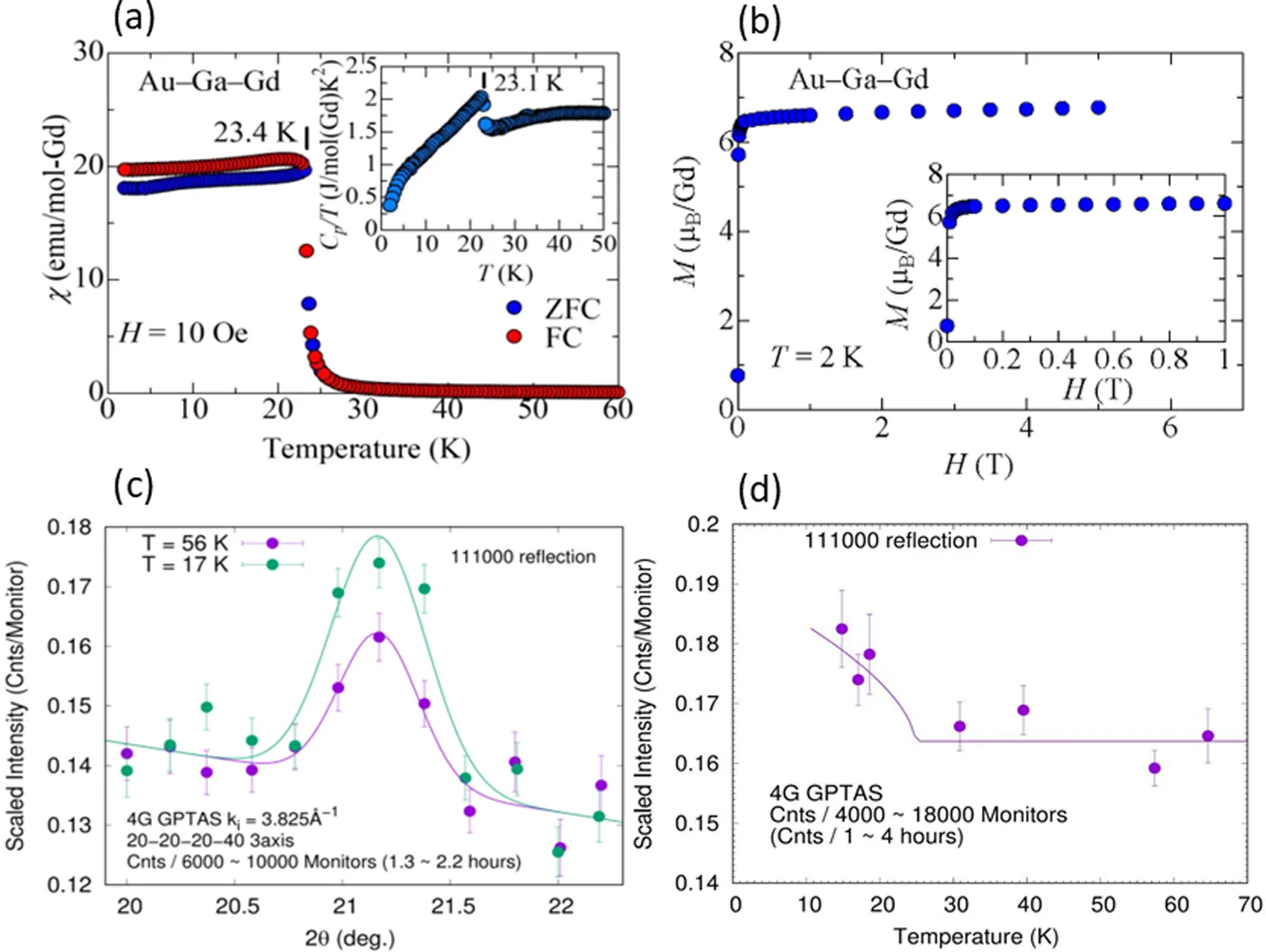
(*a*) Temperature dependences of the field-cooled (FC) and zero-field-cooled (ZFC) magnetic susceptibilities χ = *M*/*H* for Au_65_Ga_20_Gd_15_. The measurements were performed under an applied field of 10 Oe in the temperature range 2–60 K. (*b*) Magnetic-field dependences of the magnetization *M* for the same sample, measured at 2 K in the field range 0–7 T. The insets show the low-field region, highlighting the initial magnetization behaviour. (*c*) The peak profile around the [111000] reflection of the Au_65_Ga_20_Gd_15_ iQC, measured at *T* = 17 and 56 K, *i.e.* below and above *T*_C_ = 23 K. The lines serve as guides to the eye. (*d*) The temperature dependence of the integrated intensity of the [111000] magnetic reflection measured at 2θ ≃ 21.2°.

In the case of the Au_65_Ga_20_Gd_15_ iQC [Fig. 7 ▸(*a*)], a clear divergence below *T*_C_ = 23 K, indicative of an FM transition, is observed in the magnetic susceptibility. This QC is magnetically distinct from its Au_65_Ga_20_RE_15_ (RE = Tb and Dy) counterparts owing to the isotropic nature of Gd (*J* = *S* = 7/2, *L* = 0), where orbital angular momentum is quenched (referred to as a Heisenberg QC). Therefore, unlike the non-Heisenberg counterparts with RE = Tb and Dy, the field-dependent magnetization [Fig. 7 ▸(*b*)] saturates rapidly to nearly 7 μ_B_ at fields as low as ∼0.01 T, confirming that essentially all Gd spins participate in FM order. This reflects a Heisenberg nature of the spins, free from the constraints on spin orientation imposed by single-ion anisotropy. Owing to this isotropy, a global degree of freedom for spin rotation is preserved, resulting in soft magnetic behaviour with a narrow hysteresis loop.

Despite the exceptionally strong neutron absorption of natural Gd, a clear increase in the intensity of the [111000] reflection is observed below *T*_C_ [Fig. 7 ▸(*c*)], which is consistent with the FM long-range order detected in the bulk susceptibility. Moreover, the temperature dependence of the [111000] peak intensity [Fig. 7 ▸(*d*)] exhibits a clear rise below *T*_C_. Although only a single magnetic peak could be observed, it appears below the macroscopic *T*_C_ and at a position that exactly matches the 6D [111000] index. This provides solid evidence for the formation of FM order in the Au_65_Ga_20_Gd_15_ iQC.

**Figure 8:**
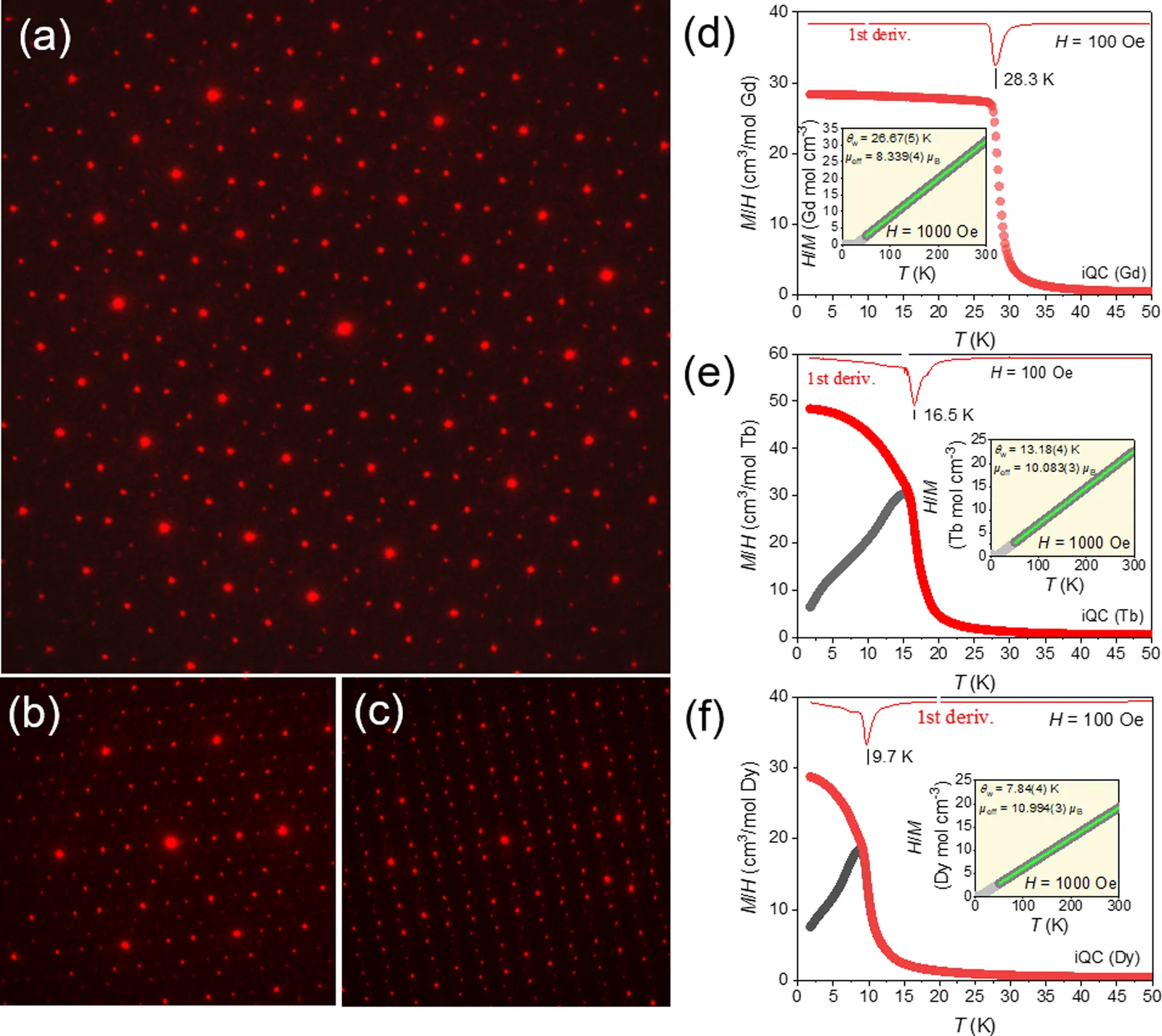
Selected-area electron diffraction patterns of the annealed Au_57.5_Cu_5.5_Al_10.5_In_12_Tb_14.5_ iQC taken along the (*a*) fivefold, (*b*) threefold and (*c*) twofold symmetry axes. Sharp fundamental reflections accompanied by numerous well defined higher-order spots demonstrate a high degree of long-range quasiperiodic coherence in the bulk state. Compared with previously reported rapidly quenched ferromagnetic iQCs, the present patterns exhibit significantly enhanced coherence, directly confirming that annealing stabilizes long-range quasiperiodic order in bulk samples without rapid quenching. (*d*)–(*f*) The temperature dependence of *M*/*H* for (*d*) Au_54_Cu_7.5_Al_12_In_12_Gd_14.5_, (*e*) Au_57.5_Cu_5.5_Al_10.5_In_12_Tb_14.5_ and (*f*) Au_57.5_Cu_5_Al_13_In_10_Dy_14.5_ iQCs measured under zero-field-cooled (ZFC, grey) and field-cooled (FC, red) conditions. Clear divergences indicate ferromagnetic transitions. The insets show the inverse susceptibility *H*/*M* over the temperature range 1.8–300 K, revealing Curie–Weiss behaviour with positive Weiss temperatures. Fits to the Curie–Weiss law yield temperature-independent susceptibilities of χ_0_ = 1.18 (2) × 10^−4^, 2.31 (2) × 10^−4^ and 4.15 (3) × 10^−4^ cm^3^ mol^−1^ for the Gd-, Tb- and Dy-based iQCs, respectively. The images are reproduced with permission from Tamura *et al.* (2026 ▸), copyright (2026) The American Chemical Society.

More recently, the discovery of bulk annealable FM iQCs in the Au–Cu–Al–In–RE (RE = Gd, Tb, Dy) system has further expanded the family of magnetically ordered QCs and overcome a major limitation of the earlier rapidly quenched compounds (Tamura *et al.*, 2026 ▸). These quinary iQCs can be synthesized by conventional arc melting followed by annealing, resulting in significantly improved quasiperiodic structural coherence and thermal stability [shown by sharp reflections in the selected-area electron diffraction patterns in Figs. 8 ▸(*a*)–8 ▸(*c*)]. Detailed investigations revealed clear FM transitions at *T*_C_ = 28.3, 16.5 and 9.7 K for the Gd-, Tb- and Dy-based iQCs, respectively [Figs. 8 ▸(*d*)–8 ▸(*f*)], accompanied by positive Weiss temperatures and λ-type specific-heat anomalies. The high structural quality of these materials enabled the first reliable quantitative study of magnetic criticality in FM iQCs, revealing element-dependent critical behaviour that ranges from nearly mean-field-like transitions in the Tb and Dy systems to non-mean-field behaviour in the isotropic Gd analogue.

It is worth noting that, at present, the microscopic details about the magnetic structures of these FM QCs remain unknown. Their determination will require the development of advanced analytical methods capable of solving magnetic structures within the framework of higher-dimensional crystallography. This remains an important challenge for future studies.

### 2.5. Case study 2: discovery of an antiferromagnetic quasicrystal

Another important case study is the recently discovered AFM order in the Au_56_In_28.5_Eu_15.5_ QC (Tamura *et al.*, 2025 ▸). This QC belongs to the Heisenberg-type Tsai family because Eu exists in a divalent state, analogous to Gd^3+^ (*J* = *S* = 7/2, *L* = 0), resulting in nearly isotropic magnetic moment behaviour. However, unlike most previously discussed systems, this compound does not follow the universal θ_W_/*d*G–*e*/*a* phase diagram shown in Fig. 4 ▸. This finding provides another example of the limitations of the *e*/*a* scale for universally describing magnetic ground states in Tsai-type compounds. Generally, Eu-based Tsai-type systems appear to deviate systematically from the previously discussed universal trend of θ_W_/*d*G–*e*/*a* shown in Fig. 4 ▸.

**Figure 9:**
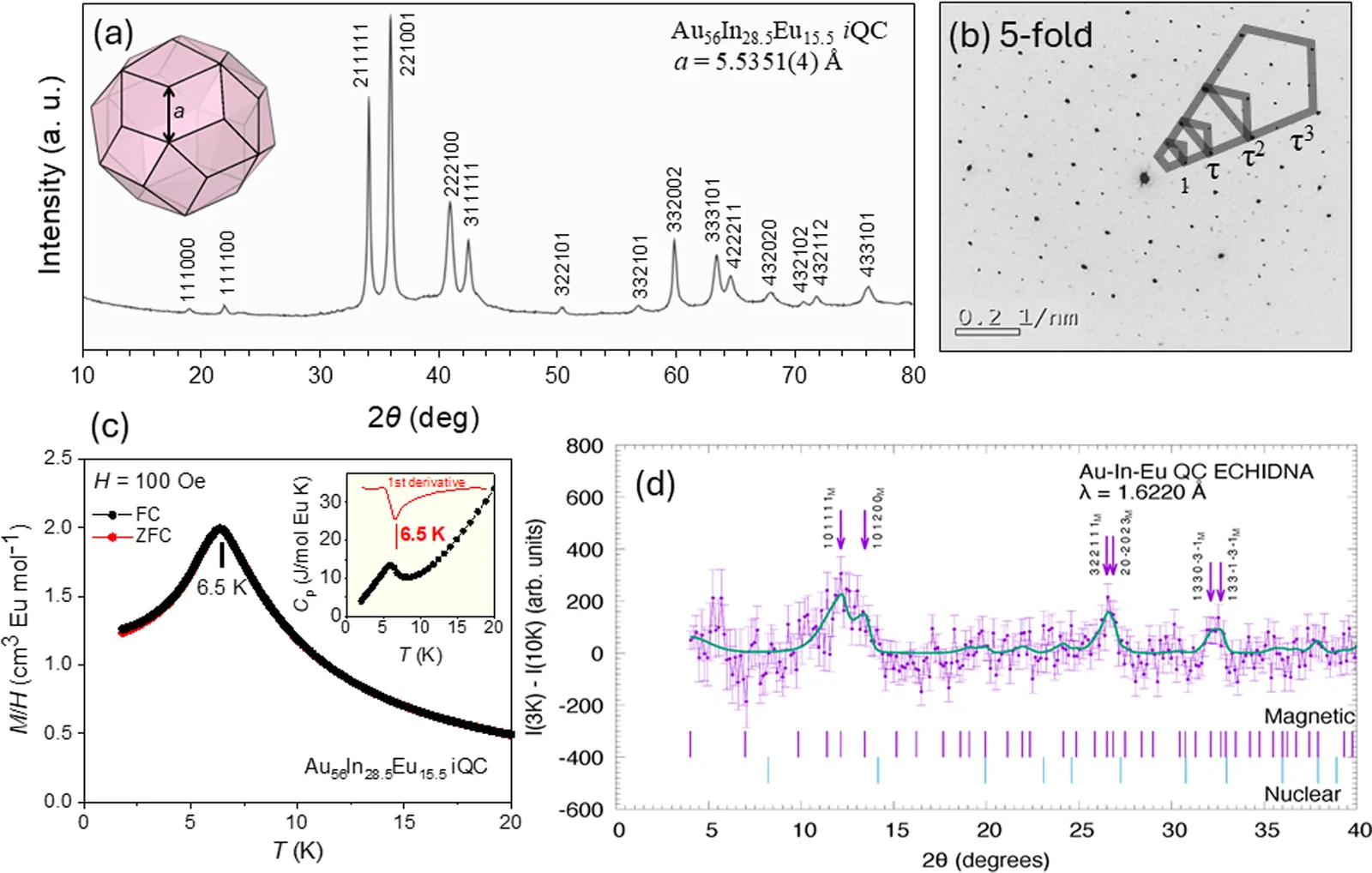
(*a*) The powder X-ray diffraction pattern of the Au_56_In_28.5_Eu_15.5_ iQC. (*b*) The selected-area electron diffraction pattern taken along the fivefold axis. The τ-scaling rule observed for the intense Bragg reflections confirms the formation of a primitive iQC. (*c*) The temperature dependence of the magnetic susceptibility (*M*/*H*) of Au_56_In_28.5_Eu_15.5_ below 20 K under ZFC and FC conditions. The inset shows the specific heat *C*_p_ of the same compound as a function of temperature, together with its temperature derivative d*C*_p_/d*T*. (*d*) The difference profile *I*(*T* = 3 K) − *I*(*T* = 10 K) in the low-angle region (2θ < 40°) of the neutron powder diffraction patterns. The blue vertical solid lines at the bottom indicate the positions of nuclear Bragg reflections expected for the icosahedral phase, indexed using 6D indices with |*Q*_⊥_| < 0.74 Å^−1^. The lattice constant (edge length of the 3D Penrose lattice) is *a* = 5.5351 (4) Å at *T* = 10 K. Violet solid lines represent the 6D indices with a doubled (magnetic) unit cell (|*Q*_⊥_| < 0.36 Å^−1^), both with *a* = 5.5351 Å. In total, three magnetic peaks (indicated by the arrows) were detected.

The Au_56_In_28.5_Eu_15.5_ QC is synthesized via arc-melting without post-processing. In the powder XRD pattern [Fig. 9 ▸(*a*)], all the diffraction peaks can be indexed as those of a P-type iQC with a quasilattice constant *a*_6D_ = 7.89 (1) Å, indicating the formation of a single-phase QC. An electron diffraction pattern taken along the fivefold symmetry axis showcasing a τ-scaling rule (where τ is the golden mean) [Fig. 9 ▸(*b*)] provides clear evidence of the intrinsic icosahedral symmetry of Au_56_In_28.5_Eu_15.5_. These results indicate that the Au_56_In_28.5_Eu_15.5_ QC belongs to the Tsai-type family and is isostructural with the prototype Cd_5.7_Yb P-type iQC (Takakura *et al.*, 2007 ▸).

In terms of magnetic susceptibility, the sample exhibits a Néel temperature of *T*_N_ = 6.5 K [Fig. 9 ▸(*c*)] and a Weiss temperature of θ_W_ = 3.41 K. The positive θ_W_ indicates dominant FM interactions, consistent with the universal trend observed in the low-*e*/*a* region in Fig. 4 ▸, as discussed above. The effective magnetic moment is μ_eff_ = 7.98 μ_B_, in excellent agreement with the theoretical value for a free Eu^2+^ ion (7.94 μ_B_), confirming the divalent state of Eu in this compound. This essentially indicates the Heisenberg nature of the spins in the AFM order.

The establishment of AFM order in the Au_56_In_28.5_Eu_15.5_ QC was further confirmed by neutron diffraction [Fig. 9 ▸(*d*)]. A combined Rietveld and Pawley refinement of the diffraction patterns, indexed using 6D reciprocal-lattice vectors, was performed by assuming a doubled magnetic unit cell of (2*a*_6D_)^6^, where 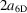 = 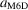 = 15.656 Å. Several magnetic Bragg reflections, including 1011111_M_, 101200_M_, 322111_M_, 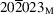, 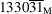 and 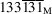 (where the subscript M denotes reflections from the doubled magnetic cell), were clearly observed [Fig. 9 ▸(*d*)]. Although a clear set of magnetic reflections was identified, the limited number of observable magnetic peaks and the high density of candidate reflections inherent to quasiperiodicity prevented a definitive determination of the magnetic structure. In particular, it remains unclear whether the AFM order is of single-*q* or multi-*q* type. Therefore, no unique magnetic propagation vector could be assigned within the present experimental resolution. The detailed magnetic modulation and structure therefore remain open questions for future investigations.

## 3. Design principle II: crystal electric field-induced anisotropy

While the valence electron concentration governs the sign and magnitude of the magnetic exchange interactions in many Tsai-type PAs, CEF effects act as a *secondary* but essential selector that determines how these interactions are geometrically realized. As described in Section 1.2, RE atoms in Tsai-type compounds occupy well defined positions on the vertices of icosahedral clusters and within AR units, which, despite being crystallographically ordered, experience low-symmetry ligand environments that generate substantial CEF effects. For RE ions with finite orbital angular momentum (non-Heisenberg ions), the CEF imposes strong single-ion anisotropy, effectively constraining each local magnetic moment to preferred symmetry-related directions. As a result, magnetic degrees of freedom in non-Heisenberg systems are not freely rotating vectors but are geometrically restricted due to the underlying icosahedral symmetry. In Tsai-type compounds, noncoplanar spin textures arise naturally as a direct consequence of the interplay between (i) icosahedral symmetry, (ii) strong single-ion anisotropy and (iii) long-range RKKY exchange interactions.

This section therefore addresses a second and complementary design principle for developing magnetic order in Tsai-type systems by controlling the CEF-induced single-ion anisotropy. After distinguishing Heisenberg and non-Heisenberg classes of RE ions, we shall discuss how the presence or absence of strong local anisotropy qualitatively changes the exchange interactions. For non-Heisenberg systems, we show that local Ising axes imposed by the CEF, combined with the icosahedral cluster geometry, naturally stabilize unconventional noncoplanar magnetic structures. In particular, the experimentally observed ‘whirling’ magnetic orders in non-Heisenberg Tsai-type 1/1 PAs are discussed as a symmetry-driven and, in this sense, designable magnetic motif. We then summarize the microscopic origin of single-ion anisotropy and the hierarchy of energy scales that determine the dominance of the CEF excitation energy scale over the RKKY interaction energy scale. Finally, we discuss magnetic orders in Heisenberg Tsai-type compounds, where CEF effects are weak and magnetic moments retain near-isotropic rotational freedom. Based on theoretical calculation results, we argue how long-range RKKY interactions on the Tsai-type cluster network can generate complex and competing magnetic textures.

### 3.1. Heisenberg versus non-Heisenberg magnetism

For RE ions with partially filled 4*f* shells and finite orbital angular momentum, such as Tb^3+^, Dy^3+^ and Ho^3+^ (electron configurations 4*f*^8^, 4*f*^9^ and 4*f*^10^, respectively, *i.e.* more than half-filled relative to the closed 4*f*^14^ shell), the CEF couples strongly to the orbital degrees of freedom. This interaction generates pronounced single-ion anisotropy, forcing the magnetic moments to align along specific local directions. As a result, these compounds are expected to display Ising-like spin behaviour and are therefore classified as *non-Heisenberg* systems. In non-Heisenberg systems, the rotational freedom of the spins is strongly restricted, giving rise to pronounced magnetic hardness in the bulk magnetic properties.

In contrast, compounds containing Gd^3+^ or Eu^2+^ ions (*J* = *S* = 7/2, *L* = 0) exhibit quenched orbital angular momentum, leading to nearly isotropic magnetic moments. Consequently, CEF-induced anisotropy becomes negligible and these materials are well described within the framework of *Heisenberg* spins, where the magnetic behaviour is governed predominantly by RKKY exchange interactions.

### 3.2. Magnetic order of periodic approximants in the presence of CEF-induced anisotropy

#### 3.2.1. Noncoplanar whirling magnetic orders

To date, microscopic magnetic structures have been experimentally resolved for a handful of non-Heisenberg Tsai-type and 1/1 PAs containing RE elements such as Tb, Dy and Ho ions. These include both FM and AFM compounds. Based on available reports, the known 1/1 PAs with resolved magnetic structures include Au_60_Ga_26_Tb_14_ (FM) (Labib *et al.*, 2024*a* ▸), Au_65_Ga_21_Tb_14_ (AFM) (Nawa *et al.*, 2023*a* ▸), Au_70_Si_17_Tb_13_ (FM) (Hiroto *et al.*, 2020 ▸), Au_70_Si_16_Tb_14_ (FM) (Gebresenbut *et al.*, 2014 ▸), Au_65.58_Si_20.78_Tb_13.64_ (FM) (Gebresenbut *et al.*, 2022 ▸), Au_69.87_Si_16.26_Tb_13.87_ (FM) (Gebresenbut *et al.*, 2022 ▸), Au_72_Al_14_Tb_14_ (AFM) (Sato *et al.*, 2019 ▸), Au_70_Al_16_Tb_14_ (AFM) (Nawa *et al.*, 2023*b* ▸), Au_61.1_Al_25.3_Ho_13.6_ (FM) (Thilakan *et al.*, 2024 ▸) and Au_68.60_Si_16.70_Ho_14.70_ (FM) (Gebresenbut *et al.*, 2022 ▸). Notably, all reported cases are limited to Au-based and 1/1 PAs. To the best of our knowledge, reliable experimental determinations of magnetic structures in non-Au-based systems, higher-order PAs or QCs are still lacking, highlighting a clear direction for future investigations. Accordingly, this subsection focuses on the available experimental studies of magnetic structures in 1/1 PAs.

**Figure 10:**
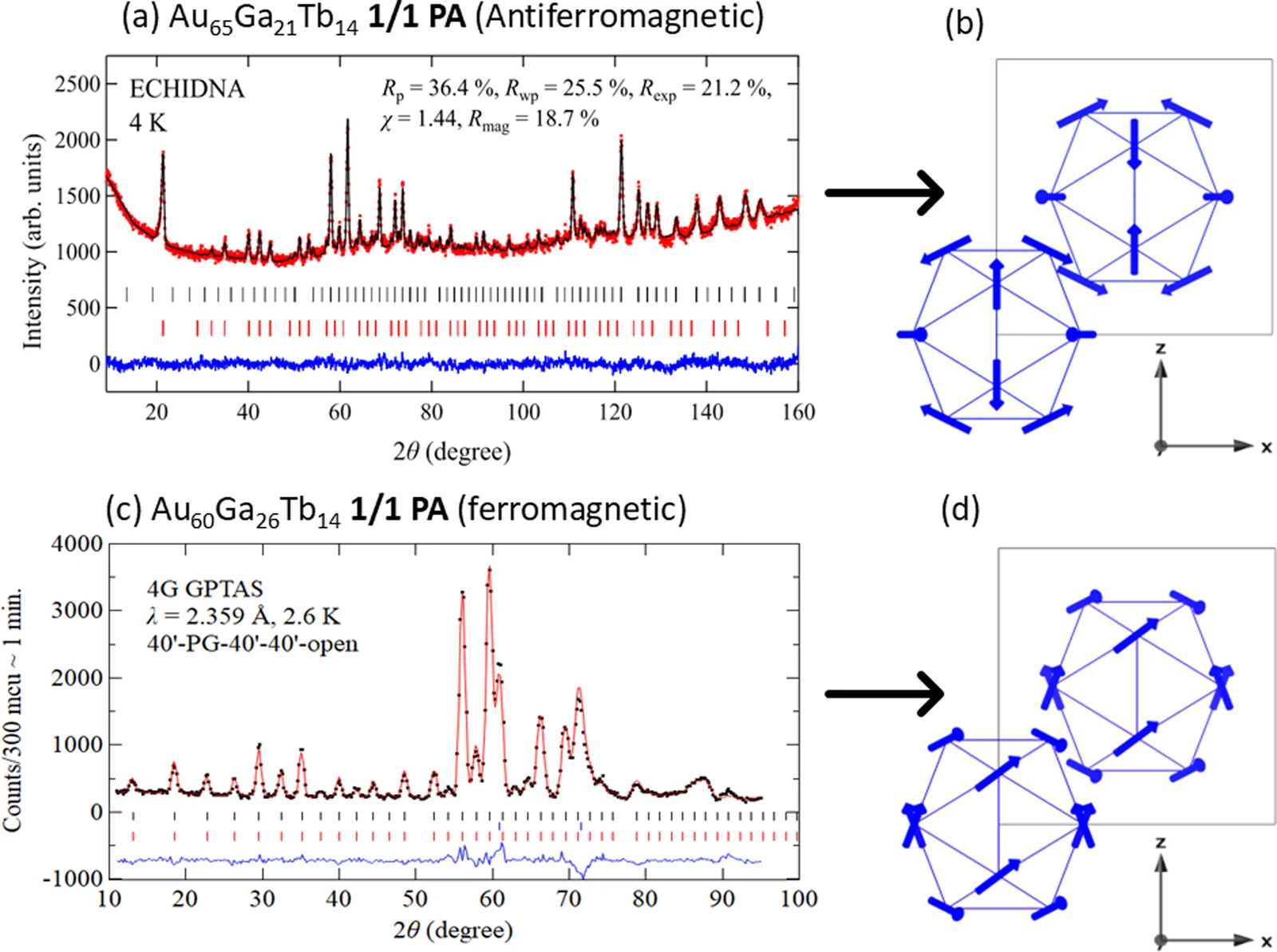
(Left) Rietveld refinement results of the neutron diffraction patterns of (*a*) AFM Au_65_Ga_21_Tb_14_ 1/1 PA (*T*_N_ = 13.2 K) and (*c*) FM Au_60_Ga_26_Tb_14_ 1/1 PA (*T*_c_ = 15.5 K) measured at base temperature. The observed intensities, calculated intensities and their difference are shown as black dots, red curves and blue curves, respectively. The positions of the nuclear reflections, aluminium reflections from the sample holder and magnetic reflections are indicated by black, blue and red vertical solid lines, respectively. (Right) Schematic representations of (*b*) the whirling AFM order in Au_65_Ga_21_Tb_14_ and (*d*) the whirling FM order in Au_60_Ga_26_Tb_14_ 1/1 PA, showing two adjacent icosahedron clusters within the cubic unit cell, viewed along the [100] crystallographic direction. After Labib *et al.* (2024*a* ▸) and Nawa *et al.* (2023*a* ▸).

Low-temperature neutron diffraction studies (below the magnetic transition temperature) of Au–SM–RE (SM = Al, Ga, Si; RE = Tb, Dy and Ho) 1/1 PAs reveal clear magnetic peaks at low angles in the diffraction patterns [see Figs. 10 ▸(*a*) and 10 ▸(*c*) for a representative AFM Au_65_Ga_21_Tb_14_ 1/1 PA (*T*_N_ = 13.2 K) and FM Au_60_Ga_26_Tb_14_ 1/1 PA (*T*_C_ = 15.5 K), respectively (Labib *et al.*, 2024*a* ▸; Nawa *et al.*, 2023*a* ▸)]. Refinement of the patterns using magnetic representation theory (Izyumov *et al.*, 1979 ▸) unveiled noncoplanar ‘whirling’ magnetic structures, represented by the magnetic space groups 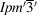 (No. 204.5, OG setting) and 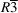 for the AFM and FM orders, respectively [see Figs. 10 ▸(*b*) and 10 ▸(*d*) for the refined AFM and FM magnetic moment configurations, respectively, on the two adjacent icosahedron clusters within the unit cells].

**Figure 11:**
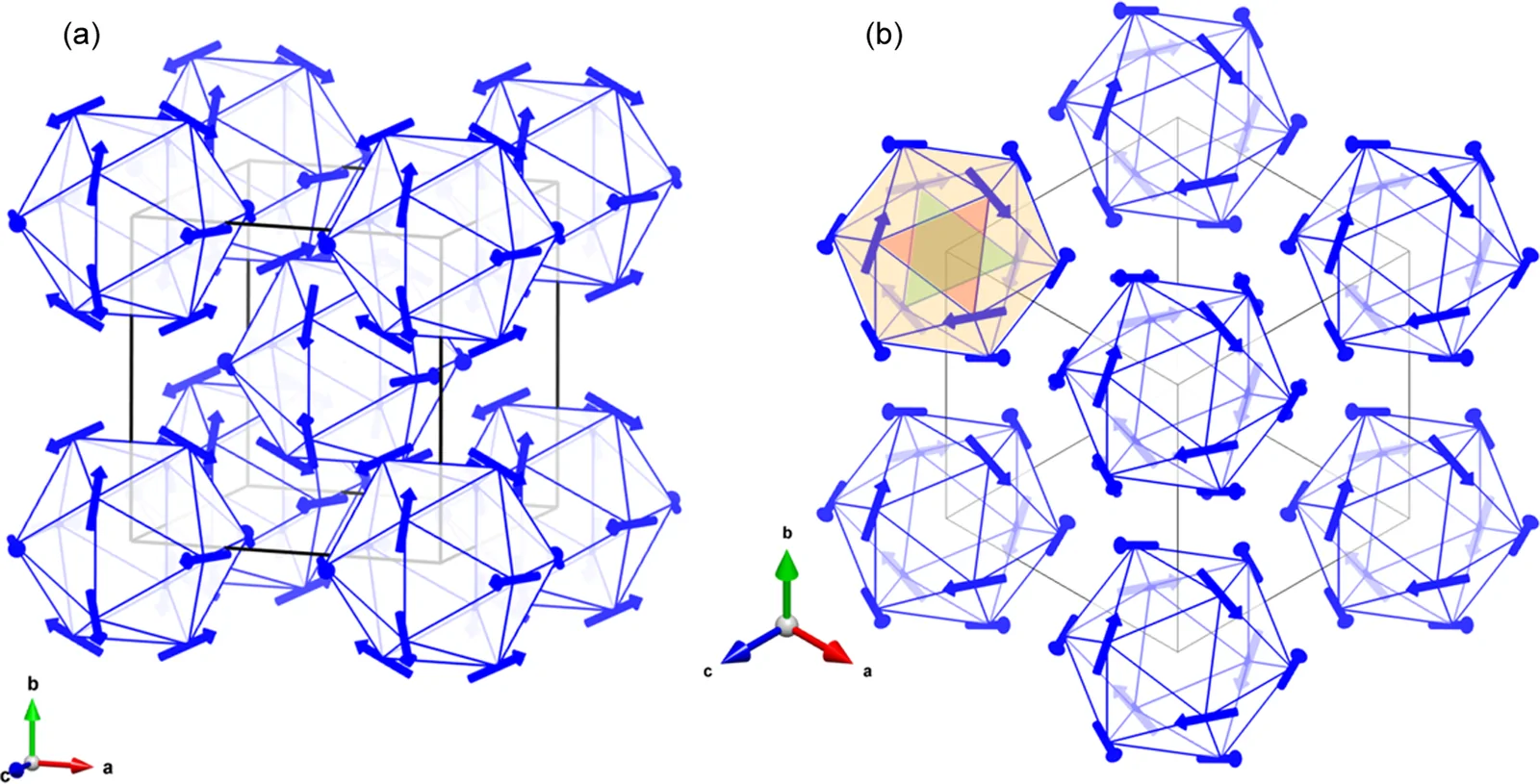
The spin configuration of the whirling AFM order on icosahedral clusters within a unit cell of a 1/1 PA, viewed along different crystallographic directions.

In these magnetic structures, threefold rotational symmetry is preserved, which produces a whirling arrangement of spins on individual icosahedral clusters when viewed along the [111] crystallographic direction [see Figs. 11 ▸(*a*) and 11 ▸(*b*) for the spin configuration of the whirling AFM order on icosahedral clusters within a unit cell of a 1/1 PA]. These structures are therefore commonly referred to as ‘whirling’ magnetic orders.

In the whirling AFM structure, spins located on opposite vertices of an icosahedral cluster are antiparallel [Fig. 10 ▸(*b*)], resulting in a zero net magnetic moment for each cluster. Likewise, equivalent sites in neighbouring clusters adopt antiparallel alignment, indicating a break of the body-centred cubic symmetry by an AFM order of cluster moments. In addition, the spins lie within the mirror plane of the icosa­hedral cluster and are oriented nearly tangentially to the cluster surface, which corresponds to a high-symmetry direction. This arrangement naturally gives rise to the characteristic whirling spin texture around the [111] axis of the body-centred cubic lattice in a 1/1 PA [Fig. 11 ▸(*b*)]. Such a configuration strongly implies that the magnetic moment orientation is constrained by the underlying icosahedral symmetry and points to the presence of CEF-induced easy-axis anisotropy along the ordered moment direction. Further evidence for strong easy-axis anisotropy, which confines the Tb^3+^ moments to their twofold (Ising-type) CEF ground state, is provided by the observation of metamagnetic transitions at finite magnetic fields (Nawa *et al.*, 2023*a* ▸; Labib *et al.*, 2024*a* ▸; Labib *et al.*, 2026*a* ▸; Labib *et al.*, 2024*b* ▸; Thilakan *et al.*, 2024 ▸; Sato *et al.*, 2019 ▸).

It should also be emphasized that, although visually reminiscent of skyrmionic textures, the whirling magnetic structure observed in the non-Heisenberg 1/1 PAs is fundamentally different from magnetic skyrmions (Mühlbauer *et al.*, 2009 ▸), where the spin rotation forms a continuous vector field covering a full 4π solid angle. In the whirling configuration, the spin chirality is not uniform but alternates between different layers. As shown in Fig. 11 ▸(*b*), the Tb moments in the upper and lower triangular layers [red and green triangles in the top left-hand icosahedron cluster in Fig. 11 ▸(*b*)] rotate in the same (clockwise) direction, whereas the intermediate buckled hexagonal layer [orange hexagon in Fig. 11 ▸(*b*)] exhibits the opposite sense of rotation. Because of this layer-dependent reversal of chirality, a well defined skyrmion number cannot be assigned to the system, as proposed for C_60_-type magnetic clusters (Coffey & Trugman, 1992 ▸).

**Figure 12:**
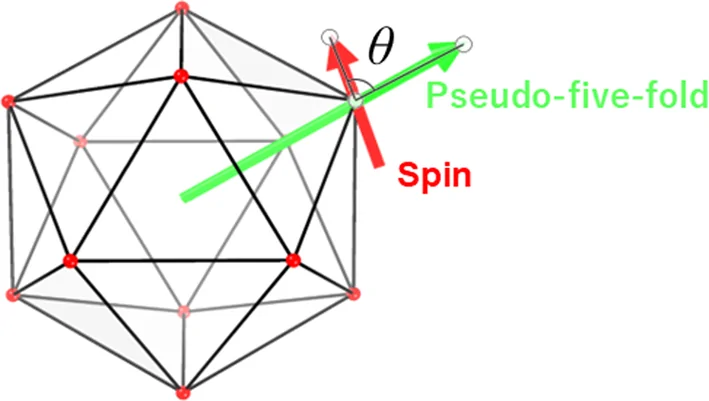
An isolated icosahedral cluster representing a canted angle of the ordered moments with respect to the pseudo-fivefold axis defined by the line connecting the cluster centre to a vertex.

Quantitative refinements of the AFM structures in the 1/1 PAs further reveal strongly canted ordered magnetic moments with respect to the pseudo-fivefold axis (defined by the line connecting the cluster centre to a vertex; Fig. 12 ▸), with canting angles of approximately 86° in Au_72_Al_14_Tb_14_ (Sato *et al.*, 2019 ▸) and Au_70_Al_16_Tb_14_ (Nawa *et al.*, 2023*b* ▸) and about 85° in Au_65_Ga_21_Tb_14_ (Nawa *et al.*, 2023*a* ▸). The corresponding ordered magnetic moment magnitudes are 7.50 (3), 7.77 (2) and 7.86 (1) μ_B_, respectively.

In contrast to the AFM orders, the whirling FM structures are characterized by parallel alignment of spins located on the opposite vertices of the icosahedral cluster [Fig. 10 ▸(*d*)]. Likewise, neighbouring clusters adopt identical spin configurations, leading to a finite net magnetic moment oriented along the [111] crystallographic direction. For Tb-based FM 1/1 PAs, the refined canting angles are approximately 85.6° for Au_60_Ga_26_Tb_14_ (Labib *et al.*, 2024*a* ▸) and 77.5° and 82.0° for Au_70_Si_17_Tb_13_ (Hiroto *et al.*, 2020 ▸) (two distinct values for the canting angles arise from the presence of multiple inequivalent magnetic sublattices). The corresponding ordered magnetic moments are about 5.6 μ_B_ and 6.9 μ_B_, respectively. In Ho-based FM 1/1 PAs, including Au_61.1_Al_25.3_Ho_13.6_ (Thilakan *et al.*, 2024 ▸) and Au_68.60_Si_16.70_Ho_14.70_ (Gebresenbut *et al.*, 2022 ▸), the canting angles fall within the range of approximately 54.8°–61.5°, accompanied by ordered magnetic moment magnitudes of ∼7.7–9.2 μ_B_.

#### 3.2.2. Microscopic origin of crystal field anisotropy in 1/1 PAs

The pronounced Ising-like anisotropy observed in whirling orders originates from the hierarchy of energy scales imposed by the single-ion CEF. For RE ions with partially filled 4*f* shells, the local CEF Hamiltonian acting on the ground-state *J* multiplet is written as 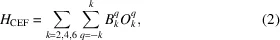
where 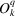 are Stevens operator equivalents and 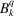 are coefficients determined by the surrounding ligand configuration. Inelastic neutron scattering measurements on the Au_70_Si_17_Tb_13_ 1/1 PA (Hiroto *et al.*, 2020 ▸) reveal a dominant CEF excitation at ℏω ≃ 4 meV, corresponding to an energy scale of ∼40 K, which is substantially larger than the exchange scale inferred from Curie–Weiss analysis (θ_W_ ≃ 10–12 K, exchange scale ∼0.1 K). A comparable separation of energy scales is also observed in the AFM Cd_6_Tb 1/1 PA (Das *et al.*, 2017 ▸). This hierarchy indicates that the orientation of Tb^3+^ moments is primarily fixed by the CEF, well before intersite exchange interactions become relevant.

Detailed analysis by Das *et al.* (2017 ▸) and Hiroto *et al.* (2020 ▸) shows that the effective CEF potential is dominated by the second-order uniaxial term 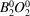 in equation (2), while higher-order contributions are strongly suppressed. Consequently, the energy required for rotating a magnetic moment away from the preferred axis is much larger than that for reversing it along the same axis. At low temperatures, the Tb^3+^ moments are, therefore, confined to two antiparallel orientations, leading to an effective Ising degree of freedom, which provides a microscopic basis for the non-Heisenberg magnetic behaviour in Tsai-type systems.

The direction of the Ising axis is determined by the low-symmetry local environment of the RE site within the Tsai-type cluster. Based on a point-charge analysis, the CEF potential can be expressed as
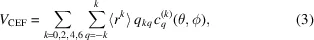
with 
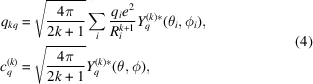
where *q*_*i*_ and (*R*_*i*_, θ_*i*_, ϕ_*i*_) denote the charge and position of the *i*th surrounding atom. The matrix elements of the spherical tensor operators are evaluated as 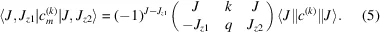


Hiroto *et al.* (2020 ▸) used statistical averaging over five thousand local atomic configurations to identify a principal anisotropy axis that lies nearly perpendicular to the pseudo-fivefold axis of the icosahedral cluster, in quantitative agreement with the magnetic moment orientation obtained from neutron diffraction refinements. The calculated longitudinal component, 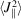 ≃ 25, corresponds to an ordered moment of ∼7.5 μ_B_, close to the experimentally refined value. A modest reduction relative to the free-ion moment is nevertheless apparent, consistent with local CEF fluctuations induced by chemical disorder. Such fluctuations are expected to broaden the excited CEF levels while preserving the ground-state pseudo-doublet and its associated easy axis. These results demonstrate that the strong single-ion anisotropy in Tsai-type 1/1 PAs is primarily encoded by the local atomic environment, whereas the RKKY interaction subsequently couples these constrained Ising-like moments to stabilize the observed noncoplanar magnetic order.

### 3.3. Magnetic order in the absence of CEF-induced anisotropy

Magnetism in Heisenberg systems is predominantly controlled by RKKY-type exchange interactions mediated by conduction electrons, while the local crystalline environment imposes only weak constraints on spin orientation. As a result, the magnetic moments are nearly isotropic, preserving an almost global rotational degree of freedom. Experimentally, this manifests as soft FM behaviour, characterized by narrow hysteresis loops and small coercive fields, typically of the order of 0.1 T or less in both QCs and PAs. Furthermore, the field dependence of the magnetization in Heisenberg systems typically reaches saturation at values close to the full free-ion moment, indicating that CEF effects are negligible. Fig. 7 ▸(*b*) shows an example of the magnetization curve of the Heisenberg FM Au_65_Ga_20_Gd_15_ iQC which saturates rapidly to nearly 7 μ_B_ at fields as low as ∼0.01 T. This is a clear sign of the Heisenberg nature of the spins, free from the constraints on spin orientation imposed by single-ion anisotropy.

Despite their central role in understanding exchange-driven magnetism in Tsai-type systems, the microscopic magnetic structures of Heisenberg compounds remain experimentally unknown. The primary difficulty stems from the exceptionally large neutron absorption cross section of Gd, which severely degrades the quality of neutron diffraction data and complicates reliable structural refinement. As a result, a definitive determination of the magnetic structure in Heisenberg QCs and PAs has not yet been achieved. Nevertheless, theoretical approaches, including Monte Carlo simulations and variational analyses, have provided valuable insight into possible magnetic orders and phase competition in these systems, as will be discussed in Section 6.

## 4. Design principle III: structural control of geometric frustration

### 4.1. Orientational order of central tetrahedron and magnetic consequences

One of the most intriguing topics, not only among QCs and PAs but also in intermetallic compounds more broadly, is the interplay between atomic structure and physical properties, particularly magnetism. Elucidating the governing principles of this relationship provides valuable guidelines for designing new magnetic states and functionalities.

As discussed above, tuning *e*/*a* serves as a powerful tool for controlling magnetic properties in a wide range of intermetallic compounds based on the Slater–Pauling framework (Kübler, 2009 ▸). Beyond *e*/*a*, additional structural degrees of freedom can be exploited in cluster-based structures, in particular QCs and PAs, to control magnetic interactions. One particularly important feature is the orientational order–disorder of the central tetrahedron unit, a phenomenon intrinsic to several known PAs.

**Figure 13:**
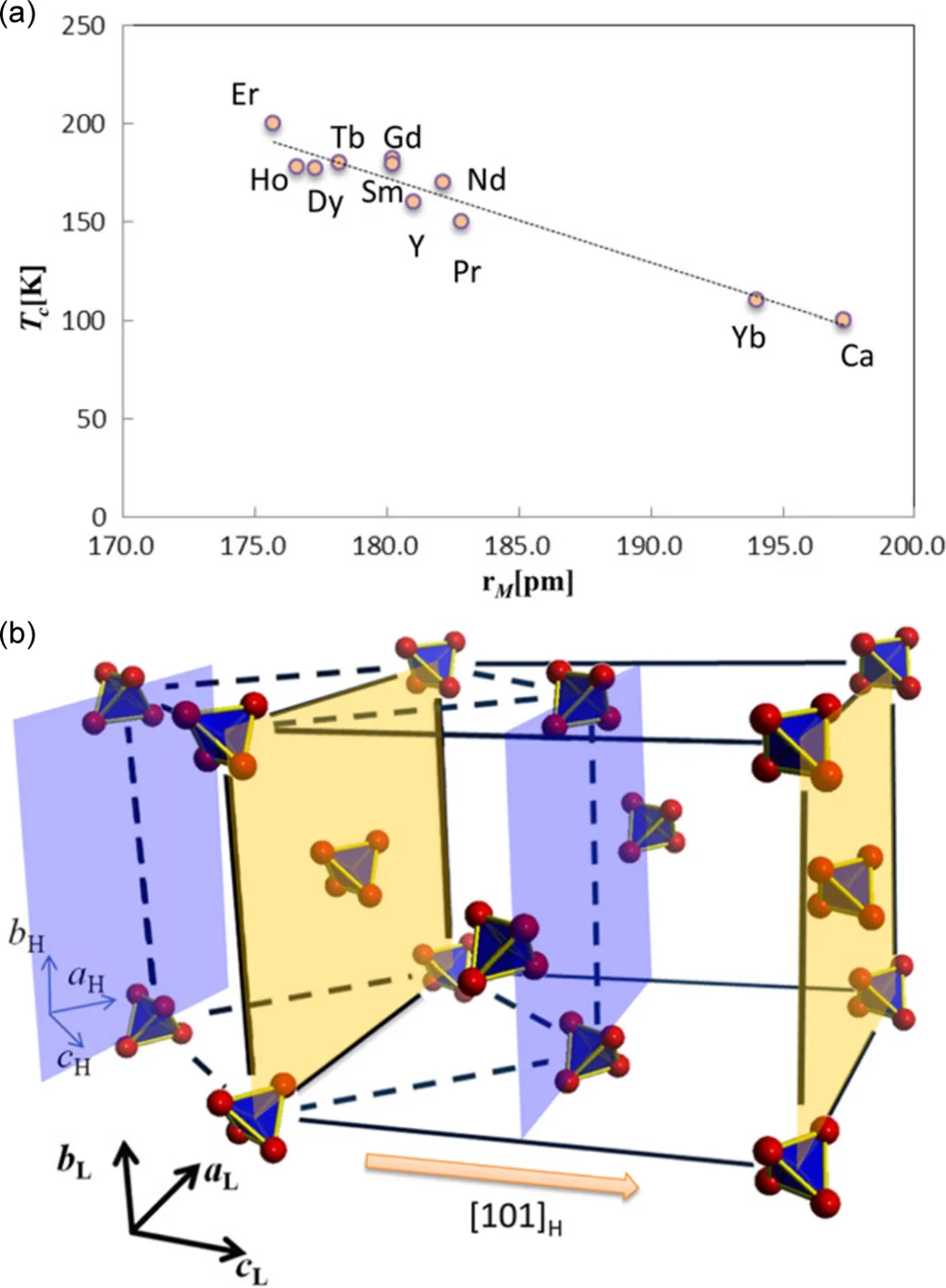
(*a*) The transition temperature (*T*_C_) of Cd_6_*M* as a function of the atomic radius *r*_*M*_ of *M*. (*b*) A schematic illustration of the low-temperature superstructure of Cd_6_*M*, showing the orientation of the Cd_4_ tetrahedron at the cluster centre (*M* = Ca, Y, Pr, Nd, Sm, Gd, Tb, Dy, Ho, Er and Tm) with respect to the Tsai-type cluster. Planes of different colours represent distinct orientations of the Cd_4_ tetrahedra. Adapted from Nishimoto *et al.* (2013 ▸).

For example, in Cd_6_RE 1/1 PAs, orientational order of the central tetrahedron occurs naturally around 100–200 K (depending on the RE element) (Nishimoto *et al.*, 2013 ▸), breaking the cubic symmetry. Fig. 13 ▸(*a*) presents the variation of transition temperature *T*_C_ in Cd_6_*M* (*M* = Ca, Y, Pr, Nd, Sm, Gd, Tb, Dy, Ho, Er and Tm) 1/1 PAs as a function of the atomic radius (*r*_*M*_) of *M*. In Fig. 13 ▸(*b*), a schematic illustration of the low-temperature superstructure of Cd_6_*M* is shown. This structural order induces a subtle lattice distortion that is believed to provide partial relief of the magnetic frustration inherent to the nearly perfect icosahedral arrangement of local magnetic moments, thereby inducing the AFM order in these compounds (Mori *et al.*, 2012 ▸; Tamura *et al.*, 2010 ▸).

**Figure 14:**
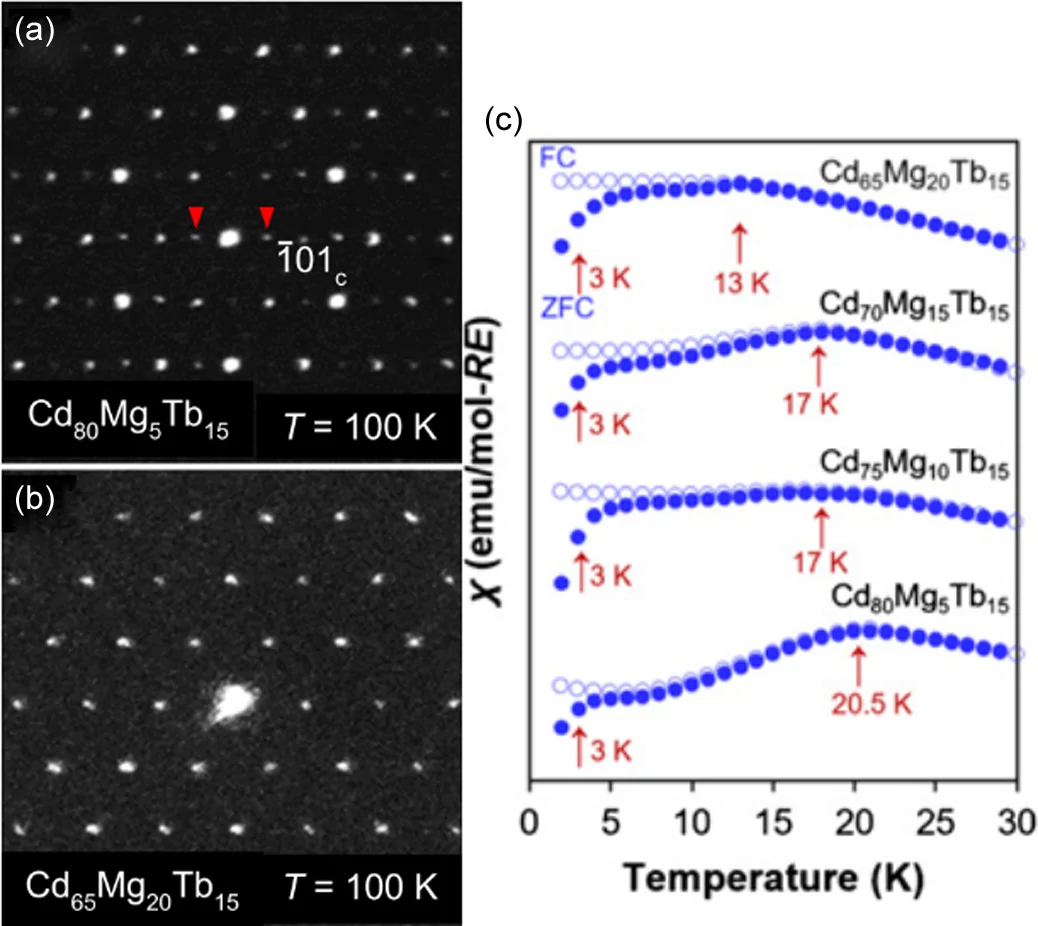
(*a*, *b*) Selected-area electron diffraction patterns taken along the [111] incidence for Cd_80_Mg_5_Tb_15_ and Cd_65_Mg_20_Tb_15_ at 100 K. Superlattice reflections are observed in Cd_80_Mg_5_Tb_15_, as indicated by arrowheads. (*c*) Magnified low-temperature direct current magnetic susceptibilities of the 1/1 PAs containing 5, 10, 15 and 20 at.% Mg in the Cd–Mg–Tb system. Adapted from Labib *et al.* (2020*b* ▸).

Experimental support for this conjecture has been provided by low-temperature transmission electron microscopy studies on Cd_85−*x*_Mg_*x*_Tb_15_ (*x* = 5, 10, 15, 20) 1/1 PAs (Labib *et al.*, 2020*b* ▸), where the AFM order disappears when superstructure reflections vanish upon Mg addition beyond 10 at.% (Fig. 14 ▸), directly linking the orientational order of the cluster centre unit to the long-range magnetic stability.

**Figure 15:**
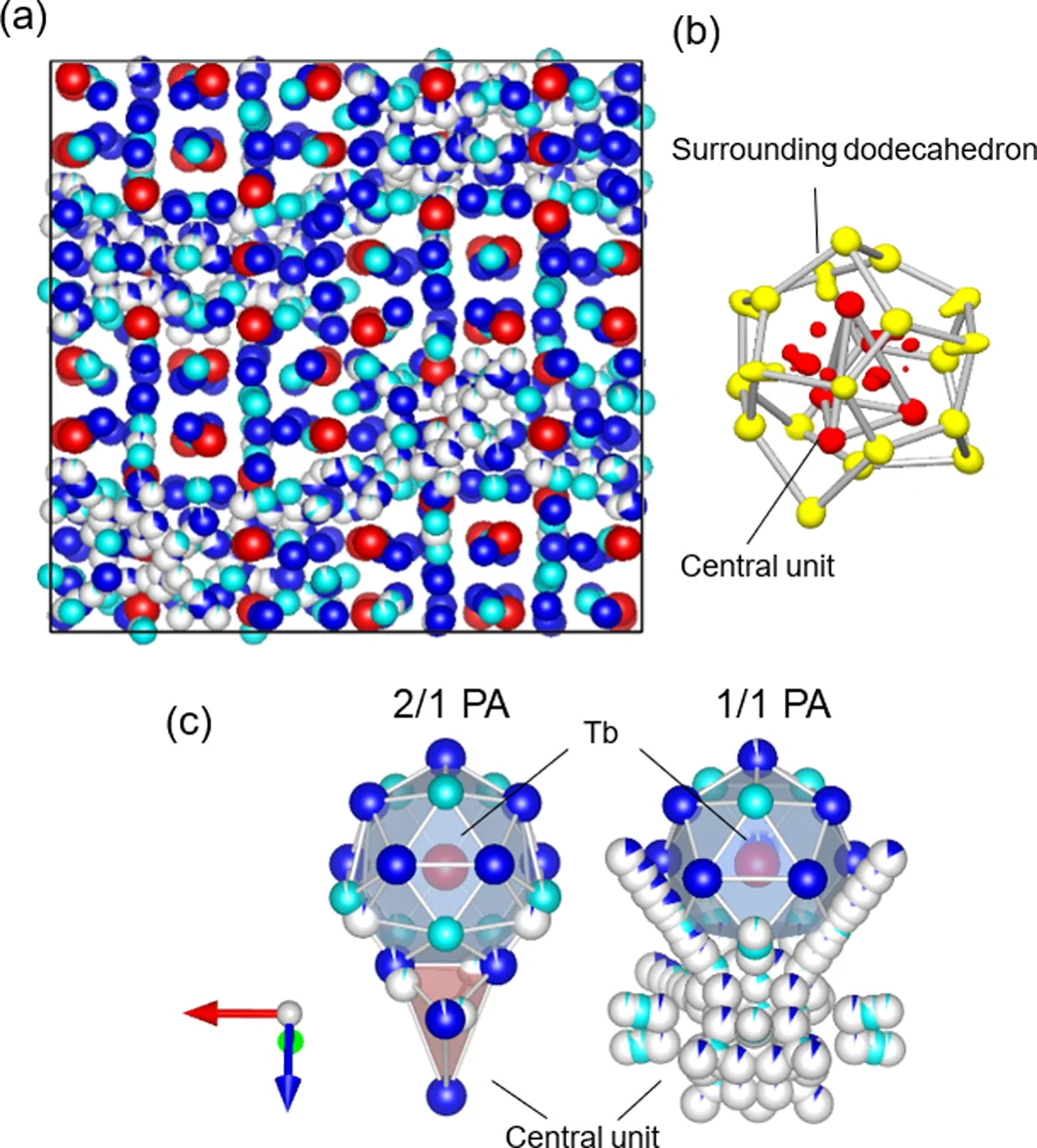
(*a*) The crystal structure of the Ga_50_Pd_35.5_Tb_14.5_ 2/1 PA unit cell with a lattice parameter of 23.1449 Å. Ga, Pd and Tb atoms are shown in dark blue, light blue and red, respectively. (*b*) Electron-density iso­surfaces derived from *F*_obs_ at the 18 e Å^−3^ level, highlighting the cluster core (red) and the distorted dodecahedral shell (yellow). (*c*) A comparison of the Tb local coordination environments in the Ga_50_Pd_35.5_Tb_14.5_ 2/1 PA and the corresponding 1/1 PA. Adapted from Labib *et al.* (2023 ▸).

Further evidence for the interconnection between the orientational order of the central tetrahedron and the magnetism is provided by structural analysis of the AFM Ga_50_Pd_36_Tb_14_ 2/1 PA (Labib *et al.*, 2023 ▸). In the atomic structure of this particular 2/1 PA, two key features are identified: (i) exceptionally low chemical disorder, particularly around Tb^3+^ ions, and (ii) an orientationally ordered trigonal pyramid at the cluster centre, which distorts surrounding shells [Figs. 15 ▸(*a*) and 15 ▸(*b*)]. In contrast, the corresponding 1/1 PA with identical chemical composition lacks such ordering and exhibits spin-glass behaviour (Labib *et al.*, 2022*b* ▸) [Fig. 15 ▸(*c*)]. These observations indicate that orientational order of the tetrahedron plays a crucial role in stabilizing AFM order.

In some 1/1 PAs such as Cd_37_Ce_6_ (Gómez & Lidin, 2003 ▸), the central tetrahedron is originally ordered even at room temperature (space group 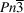). In this 1/1 PA, four of the eight Cd_8_ cubes connecting neighbouring clusters are selectively occupied, inducing orientational order via chemical pressure (Labib *et al.*, 2021 ▸). Exploiting these Cd_8_ interstitial sites as a tuning parameter of magnetism appears to be a promising direction for future research.

**Figure 16:**
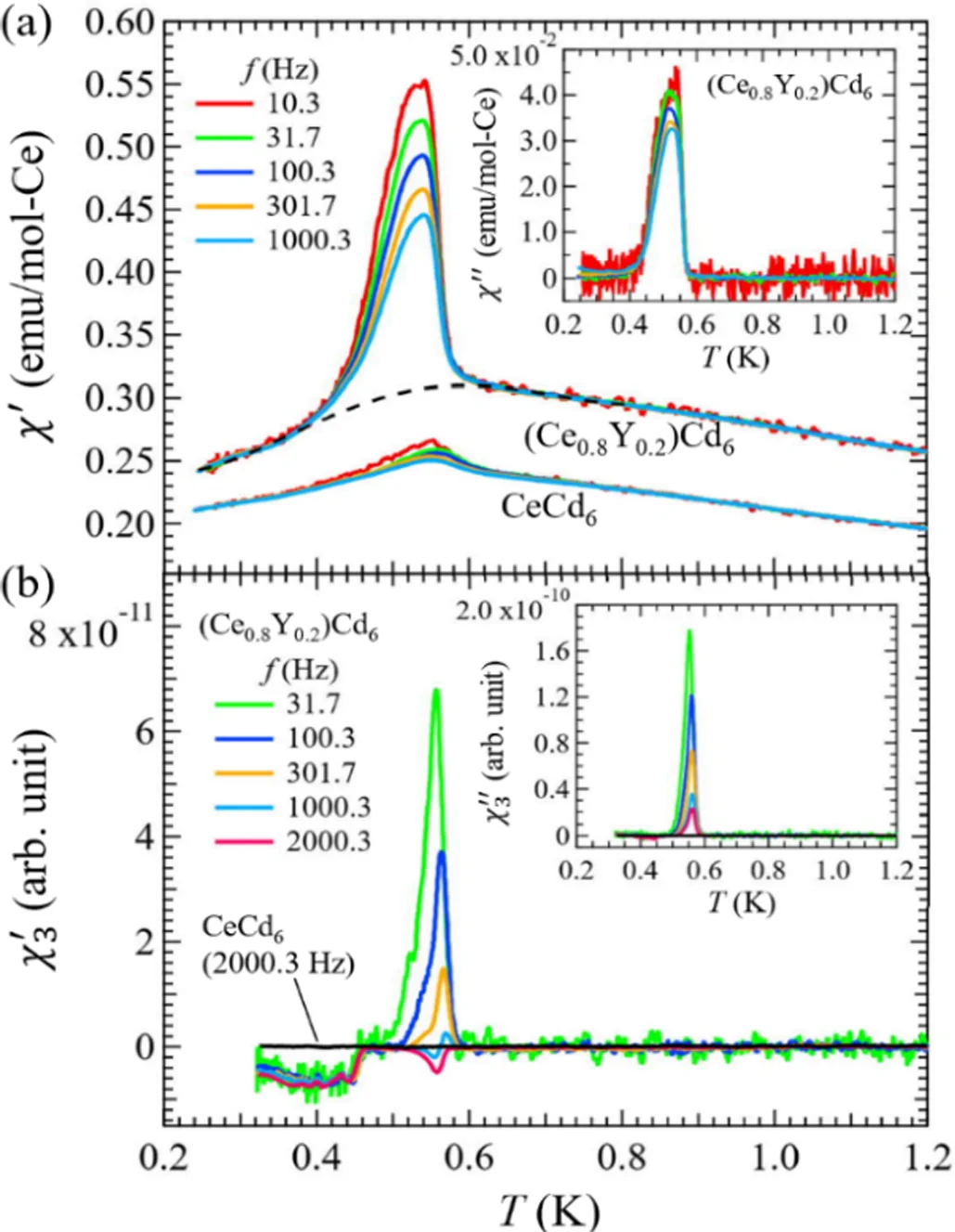
The temperature dependence of the in-phase component of the first-harmonic alternating current (ac) susceptibility χ′ for various ac frequencies. The dashed curve serves as a guide to the possible frequency-independent component. (*b*) The same for the in-phase component of the third-harmonic ac susceptibility 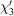; for CeCd_6_, the data at 2000.3 Hz are shown. The insets in panels (*a*) and (*b*) display the corresponding out-of-phase components. Adapted from Shiino *et al.* (2025 ▸).

A very recent study (Shiino *et al.*, 2025 ▸) reported anomalous magnetic behaviour in the dilute (Ce_0.8_Y_0.2_)Cd_6_ 1/1 PA, where Y substitution was found to suppress the orientational order of the central tetrahedron. The system does not conform to conventional static magnetic order or spin-glass behaviour. While specific-heat and resistivity measurements suggest the presence of a static magnetic component, alternating current susceptibility reveals a sharp frequency-dependent peak superimposed on a broad background signal. Furthermore, the third-harmonic susceptibility χ_3_ exhibits pronounced frequency dependence and does not follow Vogel–Fulcher behaviour, thereby excluding conventional spin-glass freezing (Fig. 16 ▸). These observations point to the coexistence of static magnetic order and slow spin dynamics, opening new perspectives on emergent magnetic phenomena in Tsai-type systems.

### 4.2. Central RE substitution

**Figure 17:**
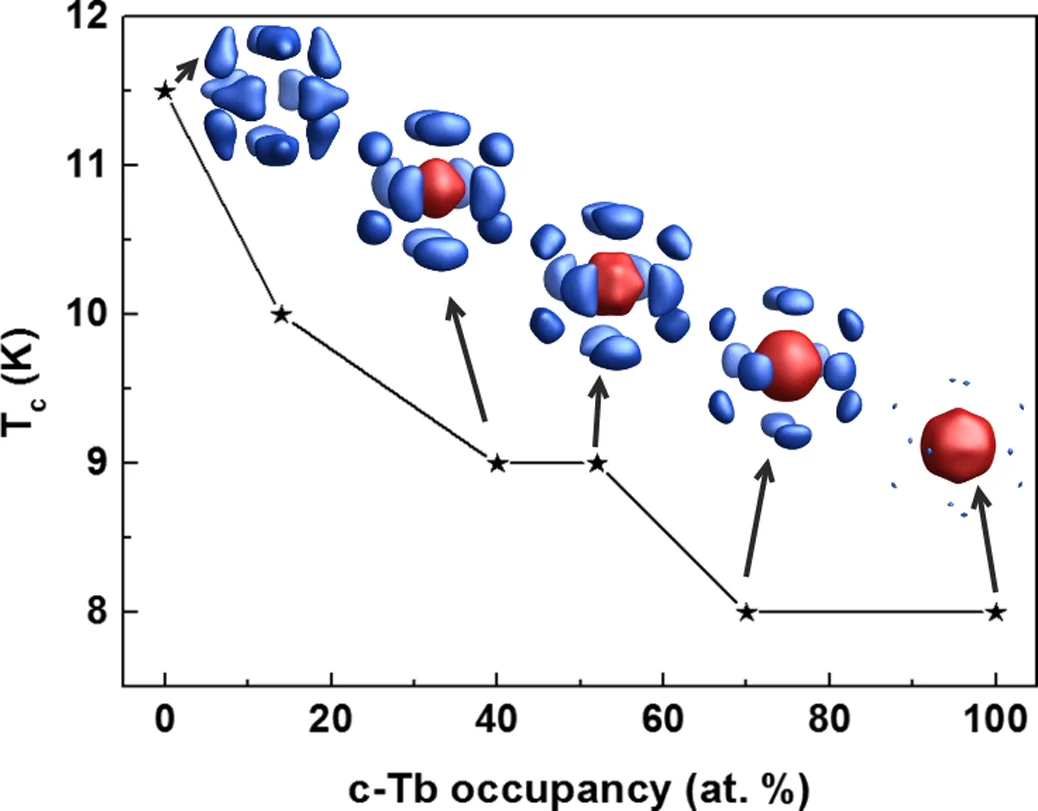
Plots of *T*_C_ versus central Tb occupancy obtained from the Au–Si–Tb 1/1 PAs. The electron-density isosurfaces near the cluster centre for the samples are provided for clarity. Adapted with permission of American Chemical Society from Gebresenbut *et al.* (2016 ▸); permission conveyed through Copyright Clearance Center, Inc.

Another structural degree of freedom in Tsai-type 1/1 PAs arises from partial or complete substitution of the cluster-centre tetrahedron by RE ions (Thilakan *et al.*, 2024 ▸; Gebresenbut *et al.*, 2016 ▸). In Tsai-type Au–(Si or Ge)–RE 1/1 PAs, in particular, the disordered tetrahedron can be partially or fully replaced by RE (Gebresenbut *et al.*, 2016 ▸). This introduces an additional magnetic moment at the cluster centre and modifies both local coordination and intercluster exchange pathways. As a result, key magnetic properties such as transition temperature, anisotropy and magnetic ground state can be tuned via central RE occupancy. Increasing RE occupancy at the cluster centre has been shown to reduce the *T*_C_ in FM systems (see Fig. 17 ▸ for the Au–Si–Tb 1/1 PA) and drive the system toward *ferrimagnetism* (Gebresenbut *et al.*, 2016 ▸). Additionally, partial substitution reduces the size of the ordered magnetic moment (Thilakan *et al.*, 2024 ▸).

## 5. Magnetism in QCs versus PAs

A direct comparison of magnetic order between QCs and PAs remains challenging, as their magnetic properties are governed by multiple interrelated parameters whose relative importance varies across alloy systems. Therefore, several representative case studies are discussed below to highlight characteristic behaviours and trends.

One important aspect concerns the comparison of magnetic frustration in QCs and PAs. In general, the degree of frustration is quantified by the frustration parameter |θ_W_/*T*_f_|, where *T*_f_ denotes the spin freezing temperature. Higher-order PAs and QCs often, but not always, exhibit lower degrees of frustration compared with 1/1 PAs. For example, |θ_W_/*T*_f_| values of 2.3 and 3.6 were reported for the Au–Al–Tm QC and 1/1 PA, respectively (Nakayama *et al.*, 2015 ▸). Similarly, values of 2.99 and 4.65 were reported for the Ga–Pd–Gd 2/1 and 1/1 PAs, respectively (Labib *et al.*, 2022*b* ▸). This reduction in frustration has been attributed to the presence of multiple inequivalent RE sites in higher-order PA structures and QCs, which generate broader distributions of interatomic distances and relax geometric constraints.

**Figure 18:**
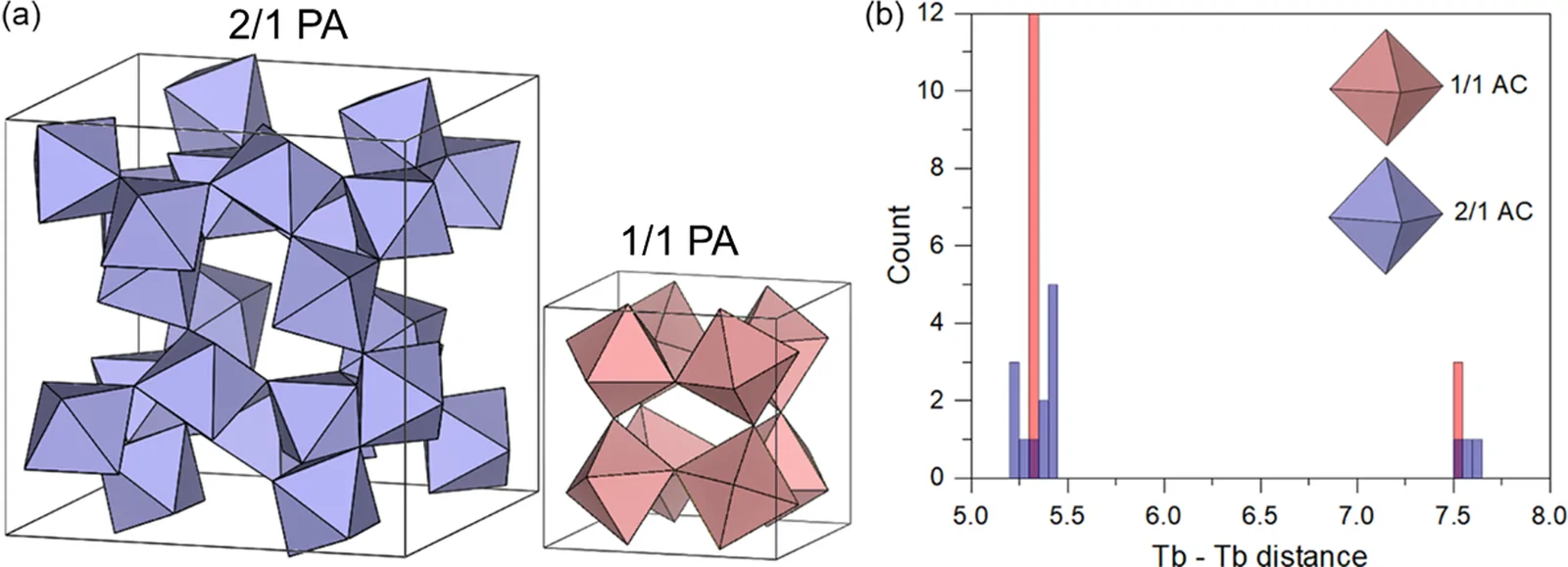
(*a*) The networks of corner-sharing tilted octahedra in the Ga_50_Pd_35.5_Tb_14.5_ 1/1 and 2/1 PAs. (*b*) A histogram of the edge-length distribution of an isolated octahedron in the two PAs. Adapted from Labib *et al.* (2022*b* ▸).

A representative example is the Ga_50_Pd_35.5_Tb_14.5_ 1/1 and 2/1 PAs, where the 1/1 PA exhibits spin-glass behaviour while the 2/1 PA shows an AFM ordered state (Labib *et al.*, 2022*b* ▸). In the 1/1 PA, the octahedral network remains nearly symmetric, with Tb–Tb distances of 5.31 Å and 7.52 Å (Fig. 18 ▸). In contrast, the 2/1 PA exhibits substantial distortion, resulting in a broader distribution of distances spanning approximately 0.35–0.55 Å. Such structural distortion is considered to provide partial relief of magnetic frustration and thereby promote the stabilization of AFM order.

**Figure 19:**
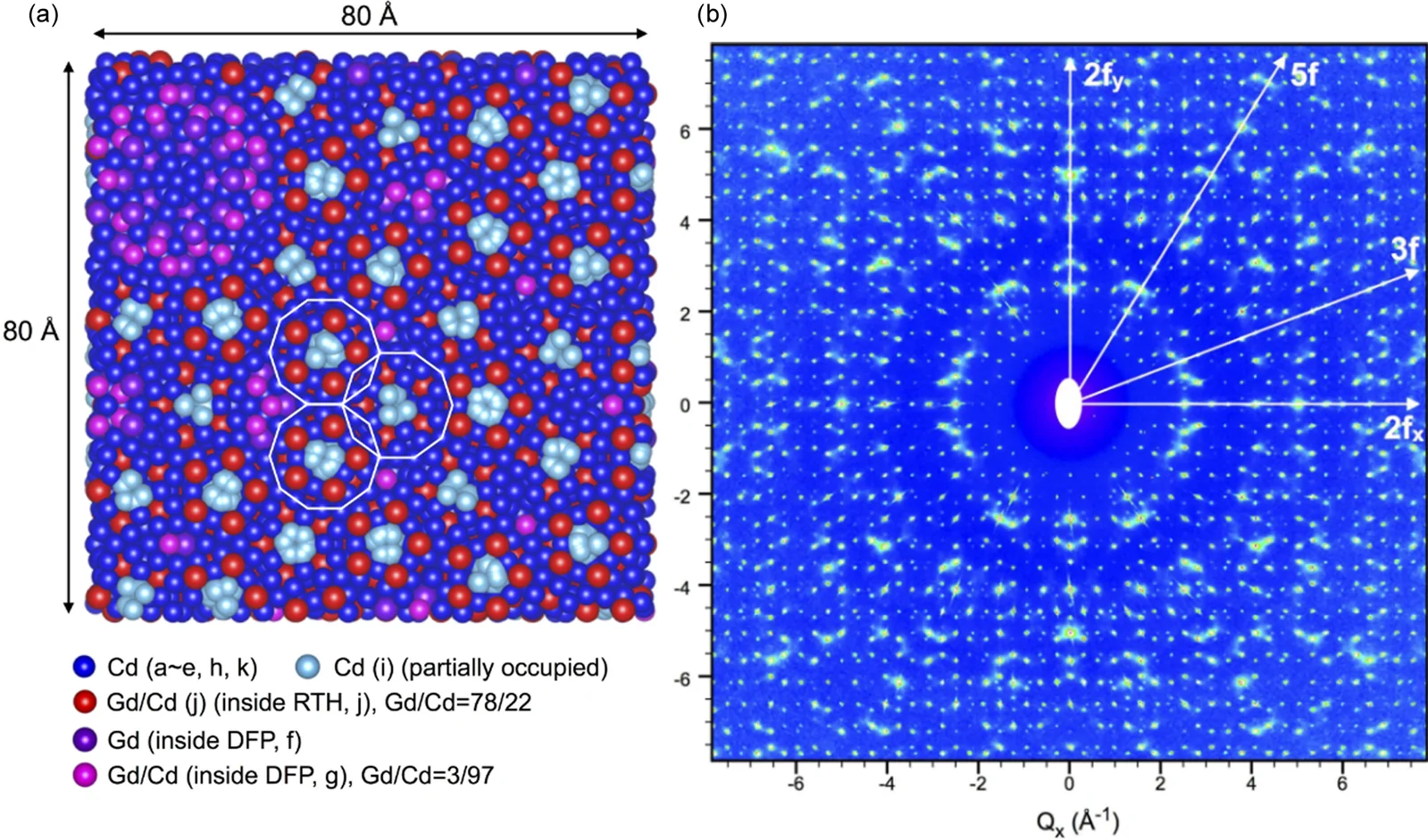
(*a*) A region of the refined structure of icosahedral Cd_7.88_Gd in the physical space normal to the fivefold direction. The white lines outline the positions of some of the Tsai-type clusters in the refined structure. (*b*) Reciprocal layer reconstruction from single-crystal X-ray diffraction of the Cd_7.88_Gd QC, showing a twofold plane up to *Q* = 7.85 Å^−1^ where streaks of diffuse scattering along the threefold direction are evident. Reprinted with permission from Yamada *et al.* (2016*b* ▸). Copyright (2016) by the American Physical Society.

The above trend, however, does not always hold. A prototype example is the observation of AFM order in the Cd_6_RE 1/1 PA series (Mori *et al.*, 2012 ▸; Tamura *et al.*, 2010 ▸), in contrast to the spin-glass-like freezing behaviour observed in the parent Cd_7.28–7.88_RE QCs (Goldman *et al.*, 2013 ▸). Typically, Tsai-type QCs contain additional RE sites within the AR units (Fig. 3 ▸), which would normally increase the RE content. However, Cd_7.28–7.88_RE (RE = Gd, Tb, Dy, Ho, Er, Tm, Y) QCs exceptionally exhibit a lower effective RE content than the 1/1 PAs, which has been attributed to chemical disorder at the RE sites. In fact, a systematic study (Yamada *et al.*, 2016*b* ▸) has shown approximately 20% chemical disorder on the RE icosahedra in the Cd_7.88_Gd QC due to partial substitution by Cd atoms [Fig. 19 ▸(*a*)]. It was therefore conjectured that such magnetic dilution of the RE icosahedra may be partly responsible for the spin-glass-like ground state observed in the binary Cd_7.28–7.88_RE (Gd, Tb, Dy, Ho, Er, Tm) QCs.

QCs also possess additional phason degrees of freedom, characteristic of aperiodic systems, giving rise to phason fluctuations, a form of intrinsic disorder that leads to phason diffuse scattering (PDS) (de Boissieu, 2012 ▸). Icosahedral Cd_7.28–7.88_RE (RE = Gd, Tb, Dy, Ho, Er, Tm, Y) QCs exhibit a substantial degree of PDS in their diffraction patterns, appearing as diffuse streaks oriented along the threefold symmetry directions, as illustrated for Cd_7.88_Gd in Fig. 19 ▸(*b*). Similar diffuse streaks elongated along the same directions have also been observed in icosahedral Zn_7.33_Sc, which can be quantitatively described by PDS with a ratio of phason elastic constants *K*_2_/*K*_1_ = −0.53, close to the threefold instability limit (Yamada *et al.*, 2016*a* ▸).

**Figure 20:**
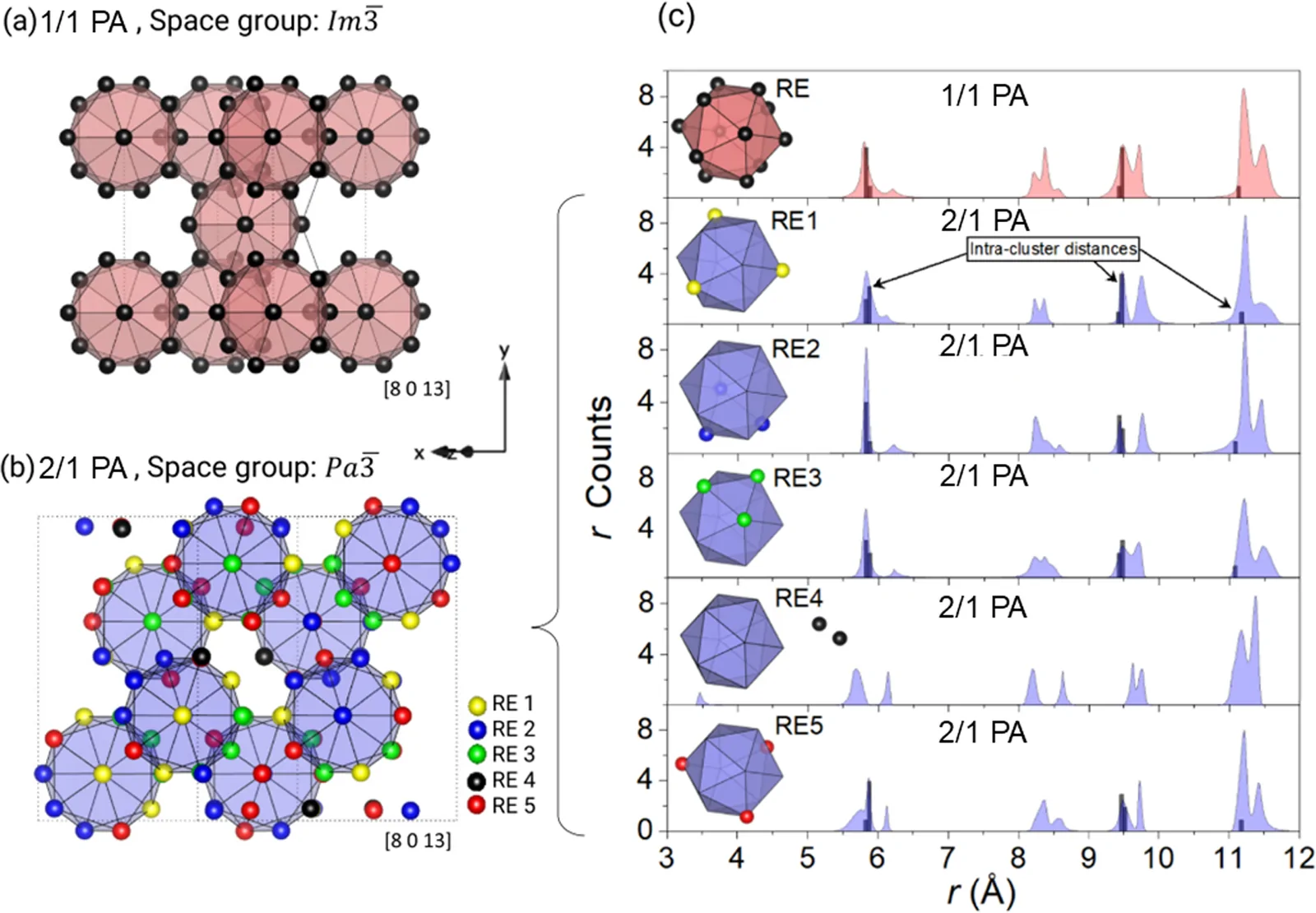
Configurations of the RE elements on the icosahedral clusters in (*a*) a 1/1 PA and (*b*) a 2/1 PA, where the different colours in (*b*) denote five symmetrically inequivalent RE sites (RE1–RE5). (*c*) The distribution of interatomic distances *r* for the 1/1 and 2/1 PAs within the range of 3–12 Å, measured from a reference RE atom to surrounding RE atoms; duplicate distances are removed and the bin size is set to 0.05 Å. For the 2/1 PA, the *r* distributions are calculated separately for each inequivalent RE site (RE1–RE5). The black bars represent intra-cluster distances within a single icosahedron composed of 12 RE moments. The top panel corresponds to the 1/1 PA, while the lower panels show the distributions for the RE1–RE5 sites in the 2/1 PA, revealing 11 distinct interatomic distances (Labib *et al.*, 2024*b* ▸).

Recently, the magnetic properties of two compositionally close quinary 1/1 and 2/1 PAs with identical *e*/*a* values close to 1.7, corresponding to the FM region in the universal *e*/*a* framework shown in Fig. 4 ▸, were systematically investigated and compared (Labib *et al.*, 2024*b* ▸). Both compounds exhibit FM order below 15.9 K and 14.2 K, respectively. Remarkably, the study revealed that the RE–RE distance distribution profiles in the 1/1 and 2/1 PAs remain highly similar over an extended range of at least 12 Å, which extends well beyond the common icosahedral cluster and includes interactions up to the third-nearest neighbours (Fig. 20 ▸). An even more intriguing result is that the RE–RE distance distribution in the 2/1 PA remains mostly similar, despite variations in RE site symmetry, particularly for the sites RE1–RE3 and RE5 located on the vertices of the icosahedral shell. This is notable because, unlike the 1/1 PA, which contains only a single crystallographically equivalent RE site, the 2/1 PA possesses five symmetrically inequivalent RE positions. The 2/1 PA also represents the closest experimentally accessible structural analogue to its parent QC, as it incorporates essentially all structural units of the QC, including the AR units that host the additional RE4 sites shown in Fig. 20 ▸.

Such similarity in the RE network suggests that, at least for the 1/1 and 2/1 PAs, substantial differences in exchange coupling energies are not expected when the electron concentration remains comparable. Whether this tendency extends fully to the parent QCs, however, remains an open question. Nevertheless, the similar sign and magnitude of θ_W_ observed across various PAs and QCs within the universal *e*/*a* framework of Fig. 4 ▸ may imply that comparable RE distance distributions may persist even in the quasiperiodic state. It is also important to note that FM interactions are generally expected to be relatively robust against minor structural variations that occur in phase transitions between PAs and QC, whereas AFM order is, in principle, much more sensitive to subtle changes in local geometry and exchange pathways. Therefore, whether similar conclusions can be extended to PAs and QCs exhibiting AFM ground states remains another open question for future investigations.

Another important insight into the stability of magnetic order in Tsai-type Cd_6_RE 1/1 PAs is provided by a dilution study reported by Shiino *et al.* (2021 ▸). Therein, slight magnetic dilution was introduced by substituting only 1–3% of the Gd atoms with nonmagnetic Y in both the prototypical Cd_6_Gd 1/1 PA and its Cd_7.88_Gd QC counterpart. Remarkably, in the 1/1 PA, even such a small level of dilution produces a drastic qualitative change in the magnetic and thermodynamic properties, including the suppression of multiple magnetic anomalies and substantial modifications of the short-range magnetic correlations. By contrast, the corresponding QC exhibits a much more gradual response, while largely retaining its spin-glass-like character. These results suggest that the long-range magnetic order realized in the Cd_6_Gd 1/1 PA exists near a delicate instability point, where coherent magnetic order emerges from a subtle balance between competing short-range correlations and weak geometric frustration. Such pronounced sensitivity to weak disorder, together with several examples of anomalous magnetic response in other Cd-based 1/1 PAs that were discussed above, further indicates that magnetic order in Cd-based Tsai-type 1/1 PAs, in general, is governed not only by the *e*/*a* ratio (see Section 2) or CEF excitations (Das *et al.*, 2017 ▸), but also by the coherence and connectivity of correlated octahedral spin networks extending beyond individual clusters (Fig. 18 ▸).

## 6. Recent theoretical advances on emergent magnetic textures in QCs and PAs

### 6.1. Point-charge analysis under CEF-induced anisotropy

#### 6.1.1. Noncoplanar spin textures in periodic approximants

A recent theoretical study (Watanabe, 2021*b* ▸) investigated magnetism in Tb-based Tsai-type 1/1 PAs using a realistic model that combines CEF-induced single-ion anisotropy with exchange interactions on Tb_12_ icosahedral clusters. The CEF was evaluated within a point-charge framework. Diagonalization of the resulting CEF Hamiltonian for Tb^3+^ (*J* = 6) revealed a well isolated ground state with strong local easy-axis anisotropy at each Tb site. The orientation of the local easy axis is parameterized by a canting angle θ with respect to the pseudo-fivefold axis (Fig. 12 ▸).

In this strong anisotropy limit, the 4*f* moments are confined to the local easy-axis directions and can be described by an effective Ising model on the icosahedron, 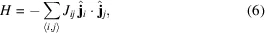
where 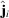 is a unit vector constrained to align parallel or antiparallel to the local easy axis at site *i*, and *J*_*ij*_ represents the exchange interaction between sites *i* and *j*. The exchange parameters were modelled using nearest-neighbour (NN, *J*_1_) and next-nearest-neighbour (NNN, *J*_2_) interactions, incorporating both intra-cluster and inter-cluster couplings. Motivated by experimental observations of positive Weiss temperatures in many magnetically ordered Tsai-type compounds, Watanabe *et al.* (2021*b* ▸) focused on a regime dominated by FM interactions (*J*_1_ > 0 and *J*_2_ > 0). This tendency is further supported by neutron diffraction studies and refined magnetic structures reported in Tb-based systems (Labib *et al.*, 2024*a* ▸).

**Figure 21:**
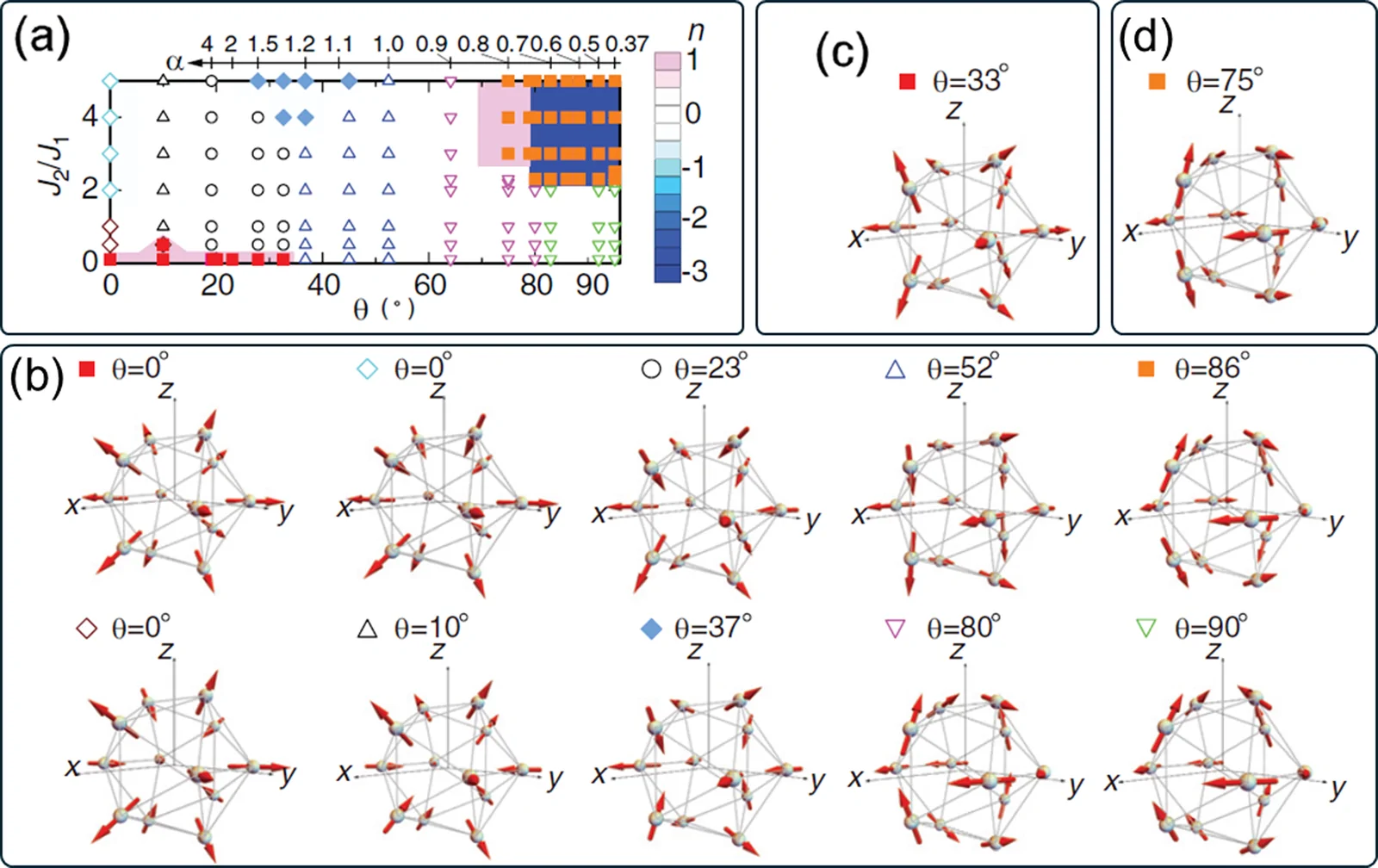
(*a*) The calculated ground-state phase diagram for the 1/1 PA based on point-charge CEF analysis. Open (filled) symbols denote ferromagnetic (antiferromagnetic) orders. A contour plot of the topological charge *n* on the icosahedron is also shown. (*b*) Magnetic textures corresponding to the symbols in panel (*a*), including the hedgehog (red square) and antiwhirling (orange square) states. (*c*) Hedgehog state for θ = 33°. (*d*) Antiwhirling state for θ = 75°, characterized by *n* = 1. Adapted from Watanabe (2021*b* ▸).

By varying the ratio *J*_2_/*J*_1_ together with the canting angle θ, a ground-state phase diagram is obtained for the Tb-based 1/1 PA (Fig. 21 ▸), revealing multiple noncollinear and noncoplanar spin configurations on the Tb_12_ icosahedron. In the phase diagram, these configurations include hedgehog, antihedgehog, whirling and antiwhirling states. These spin textures are characterized by different topological charges *n*, with the hedgehog state corresponding to *n* = 1, while the whirling-type states exhibit exceptionally large values of *n* (Watanabe, 2021*b* ▸).

#### 6.1.2. Noncoplanar spin textures in QCs

The same model discussed in Section 6.1.1 is further applied to a quasiperiodic arrangement of Tb_12_ clusters corresponding to a Cd_5.7_Yb-type QC, in which the icosahedral clusters are located at the origin and at the vertices of an icosadodecahedron and are further repeated outward in a self-similar manner with a scaling factor of τ^3^ (Watanabe, 2021*a* ▸). In this construction, the exchange interactions *J*_1_ and *J*_2_ are defined not only within each Tb_12_ icosahedron but also between neighbouring clusters. Specifically, NN and NNN interactions are assigned to Tb sites belonging to adjacent clusters connected along the bonds of the τ^3^-scaled icosadodecahedral network.

**Figure 22:**
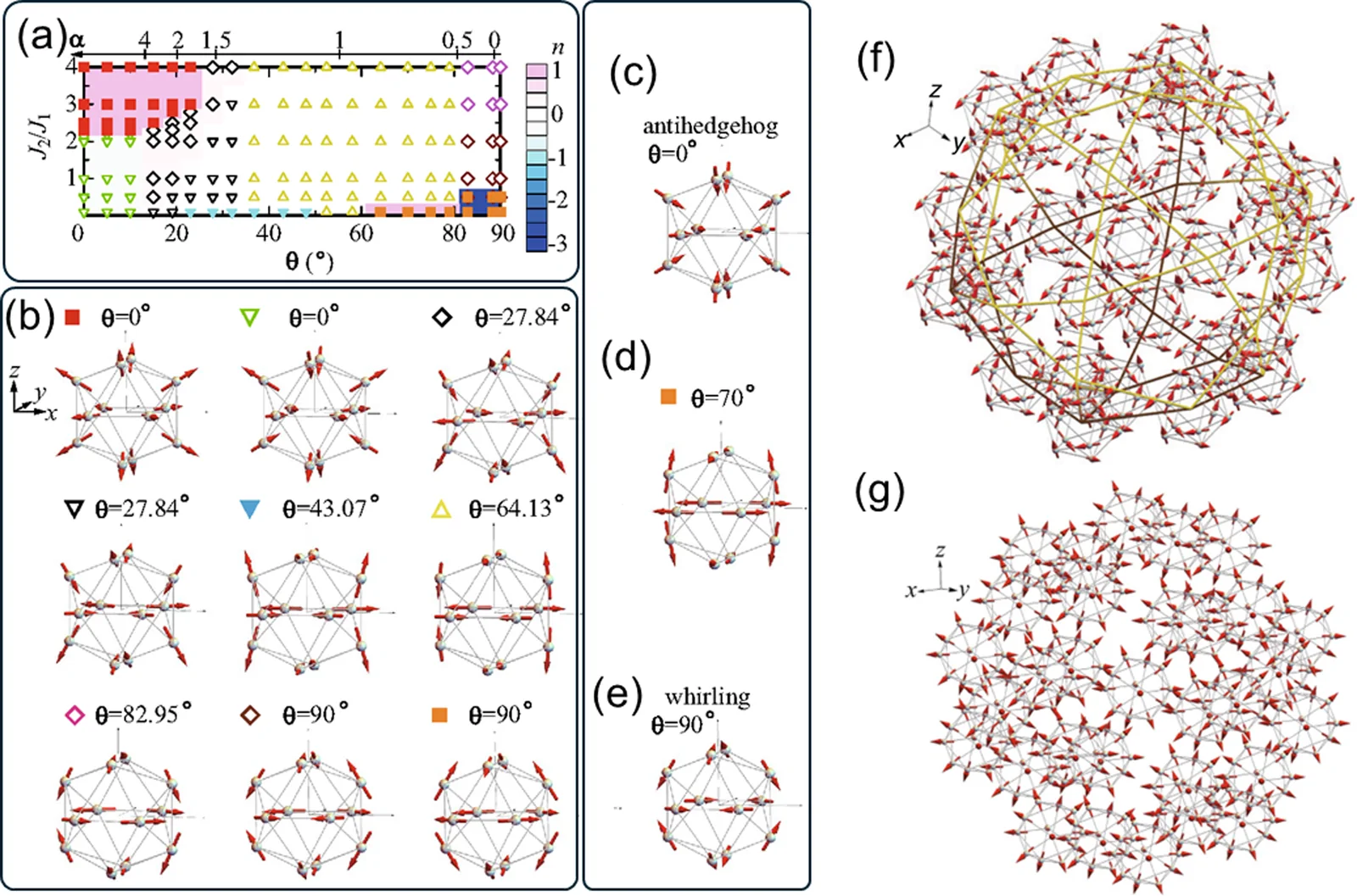
(*a*) Ground-state phase diagram of the iQC as functions of *J*_2_/*J*_1_ (*J*_1_ < 0), the canting angle θ and the ligand-valence ratio α. The contour plot of the topological charge *n* is also shown. (*b*) Magnetic textures corresponding to the symbols in panel (*a*). (*c*) Antihedgehog state for θ = 0°. (*d*) Antiwhirling state for θ = 70°. (*e*) Whirling state for θ = 90°. (*f*) The magnetic moments in the Tb_12_ clusters at the origin and at the vertices of the icosadodecahedron, shown for θ = 80°. Yellow and brown lines connect vertices on the front and back of the icosadodecahedron, respectively. (*g*) Long-range hedgehog order in the iQC for θ = 0°. Tb_12_ clusters at the origin and at the vertices of the icosadodecahedron are shown. Adapted from Watanabe (2021*a* ▸).

The magnetic ground state is obtained by evaluating the expectation value of 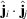 for all NN and NNN pairs, including both intra-cluster and inter-cluster contributions, and searching for the lowest-energy configuration over the full cluster network (Fig. 22 ▸). Similar to the 1/1 PAs, a wide variety of spin configurations such as the hedgehog state are realized. For the hedgehog configuration (θ = 0°) in particular, it is found that 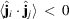 is satisfied for all such pairs, indicating that this state is energetically favoured when *J*_1_ < 0 and *J*_2_ < 0. As a consequence, the hedgehog texture formed on an individual Tb_12_ icosahedron is stabilized across neighbouring clusters, leading to a uniform long-range arrangement throughout the quasiperiodic structure.

It is worth noting that in both calculations by Watanabe performed on the 1/1 PA (Section 6.1.1) and iQC (Section 6.1.2), NN and NNN interactions are considered. The key difference is therefore not the presence or absence of such interactions, but the spatial arrangement of the Tb_12_ clusters: a periodic body-centred cubic arrangement in the 1/1 PA versus a self-similar quasiperiodic arrangement in the iQC. This difference changes which inter-cluster pairs are connected and how the same local textures are stabilized across the structure. A direct comparison between the ground-state phase diagrams of the 1/1 PA (Fig. 21 ▸) and the iQC (Fig. 22 ▸) reveals largely similar types of magnetic textures such as hedgehog, antihedgehog, whirling and antiwhirling states. This suggests that these textures are governed primarily by the local icosahedral environment and magnetic anisotropy, rather than by quasiperiodicity itself. The extent to which quasiperiodicity influences the long-range organization and collective behaviour of such magnetic textures remains an important open question for future studies.

It should also be noted that the models used in Sections 6.1.1 and 6.1.2 are minimal and do not explicitly incorporate the long-range oscillatory RKKY interactions and frustration effects expected in QC and PAs. However, as stated earlier in Section 3.2.2, the energy scale of CEF excitation is much larger (∼40 K) than the exchange scale inferred from Curie–Weiss analysis (∼0.1 K), indicating that in non-Heisenberg-based systems the orientation of RE^3+^ magnetic moments is primarily determined by CEF-induced single-ion anisotropy before RKKY exchange interactions become relevant. This probably explains why the minimal model used by Watanabe (2021*a* ▸, 2021*b* ▸) captures the experimentally observed whirling magnetic structures in non-Heisenberg systems discussed in Section 3.2.1, despite its minimal treatment of exchange interactions.

### 6.2. Beyond dipoles: multipolar magnetism in QCs

A recent theoretical study (Jeon & Lee, 2024 ▸) has revealed that icosahedral magnetic QCs can naturally host higher-rank multipolar degrees of freedom, originating from the interplay between spin–orbit coupling and CEF effects under non-crystallographic symmetry. In particular, the analysis shows that the icosahedral crystal field can stabilize Kramers doublets carrying pure magnetic octupole moments without accompanying dipolar components. This behaviour arises from the unconventional point-group symmetry of QCs, which allows crystal-field terms forbidden in periodic crystals. As a result, CEF splitting under icosahedral symmetry can produce doublets in which conventional magnetic dipole moments vanish, leaving higher-order multipoles as the primary magnetic degrees of freedom.

Beyond identifying these unconventional local degrees of freedom, Jeon & Lee (2024 ▸) stated that geometric frustration and quantum fluctuations in octupolar systems can generate highly degenerate ground states and entangled quantum phases. For AFM interactions on a single icosahedral cluster, their model yields a large manifold of degenerate configurations, which are subsequently lifted by quantum fluctuations to form nondegenerate entangled ground states. This mechanism suggests a fundamentally new design route for magnetic QCs: rather than focusing solely on dipolar magnetism controlled by RKKY exchange or anisotropy, one can engineer crystal-field environments and local symmetry to stabilize multipolar moments and explore hidden orders, quantum spin liquids and exotic Kondo phenomena. In this perspective, the non­crystallographic symmetry of QCs itself becomes a key tuning parameter for realizing unconventional quantum states.

### 6.3. RKKY-driven degeneracy and competing magnetic textures

In the absence of direct experimental evidence of magnetic structures in Heisenberg-type compounds, theoretical approaches have played a crucial role in elucidating the landscape of possible ordered states. This experimental limitation arises mainly from the large neutron absorption cross sections of Gd and Eu, which make neutron diffraction – one of the most powerful probes of microscopic magnetic structures – particularly challenging. Consequently, large-scale Monte Carlo simulations and variational energy analyses have become indispensable for understanding magnetism in these systems.

**Figure 23:**
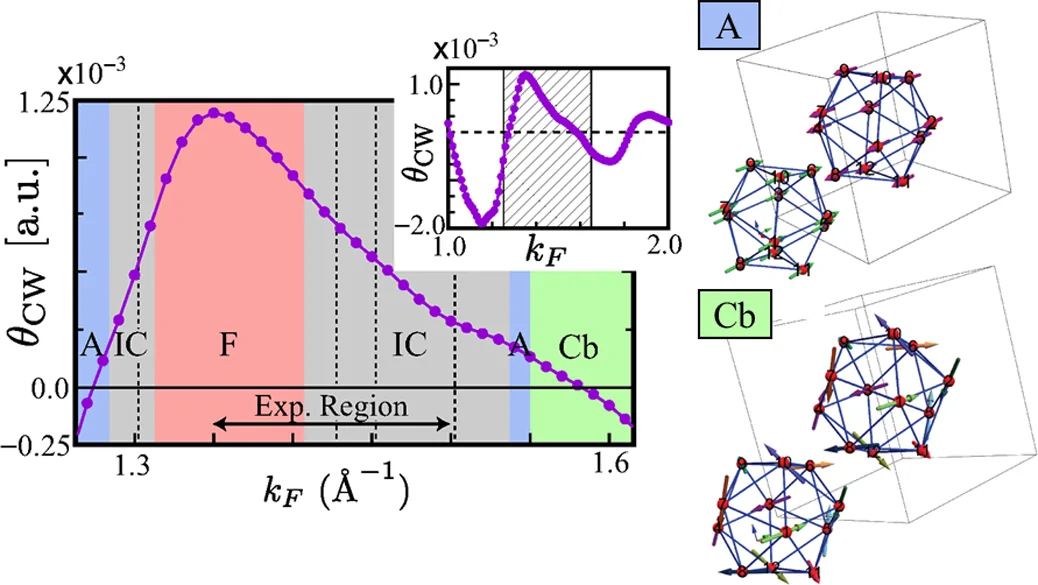
Theoretical magnetic phase diagram showing the Curie–Weiss temperature as a function of *k*_F_. The inset represents the simulated region of θ_W_. The phase diagram includes ferromagnetic (F), antiferromagnetic (A), cuboc (Cb) and incommensurate (IC) phases. Reprinted with permission from Miyazaki *et al.* (2020 ▸). Copyright (2020) by the American Physical Society.

One recent theoretical advance towards understanding magnetism in Tsai-type Heisenberg 1/1 PAs emerged from classical Monte Carlo studies of Gd-based Tsai-type 1/1 PAs (Sugimoto *et al.*, 2024 ▸; Miyazaki *et al.*, 2020 ▸), where crystal-field effects are negligible and the magnetic behaviour is governed primarily by conduction-electron-mediated inter­actions. By employing a minimal isotropic Heisenberg model with long-range oscillatory exchange couplings arising from the RKKY interaction and parameterized by the Fermi wavevector *k*_F_ (Miyazaki *et al.*, 2020 ▸), it was demonstrated that variation in *k*_F_ alone can drive the system through a rich sequence of magnetic ground states, including FM, AFM, incommensurate (IC) and cuboc (cuboctahedron, Cb) orders (Fig. 23 ▸). In the phase diagram, the FM and AFM regions are denoted by ‘F’ and ‘A’, respectively.

The resulting zero-temperature phase diagram exhibits repeated alternation between commensurate and incommensurate states, reflecting strong frustration generated by oscillatory exchange interactions on the complex icosahedral cluster network. A particularly noteworthy point is the stabilization of the cuboc phase in the large-*k*_F_ regime (corresponding to the large-*e*/*a* regime in Fig. 4 ▸), characterized by a noncoplanar spin configuration with approximately orthogonal spin orientations, vanishing net magnetization and finite vector chirality. The emergence of such a complex spin topology within a fully isotropic Heisenberg framework demonstrates that unconventional magnetic textures can arise solely from geometric complexity and long-range exchange interactions, without invoking CEF-induced anisotropy.

Building upon this framework, the same authors (Sugimoto *et al.*, 2024 ▸) recently extended the analysis by explicitly incorporating both intracluster and intercluster RKKY interactions. Their results clarified that magnetic degeneracy in Heisenberg PAs originates from competition among multiple interaction length scales, and that subtle variations in electronic filling can readily alter the energetic hierarchy among competing magnetic textures.

## 7. Critical phenomena and universality in Tsai-type compounds

While the microscopic mechanisms governing magnetic ground states in Tsai-type systems have been discussed in detail, an equally important issue concerns the nature of the magnetic phase transition itself and the associated critical behaviour near *T*_C_ or *T*_N_. Critical phenomena provide an independent and stringent test of the universality underlying the magnetic interactions introduced above. In contrast to ground-state analysis, which primarily addresses static spin configurations, the determination of critical exponents probes collective fluctuations and long-wavelength correlations that develop as the transition temperature is approached. Because universality classes are determined by symmetry, spatial dimensionality and the effective interaction range, the study of criticality offers a powerful framework to assess whether magnetism in quasiperiodic systems follows conventional 3D universality classes or instead reflects distinctive features arising from quasiperiodic geometry.

In the following, we summarize experimental determinations of critical exponents in Tsai-type compounds, and discuss their implications for the effective interaction range and universality in quasiperiodic magnetic matter.

### 7.1. Experimental critical exponents

The determination of critical exponents is essential for elucidating the nature of magnetic phase transitions in QCs. To date, however, only a limited number of QCs and PAs have been investigated from the viewpoint of critical behaviour. Most of them have employed conventional Arrott-plot analysis based on Banerjee’s criterion (Banerjee, 1964 ▸) to extract the critical exponents β, γ and δ. Despite the limited dataset, all reported results consistently indicate that the FM transitions are of second order in nature, irrespective of the underlying structure type (1/1 PA, 2/1 PA or QC) and regardless of whether the RE ions exhibit Heisenberg or non-Heisenberg character (Tamura *et al.*, 2026 ▸; Takeuchi *et al.*, 2023 ▸; Labib *et al.*, 2024*b* ▸; Labib *et al.*, 2025 ▸; Shiino *et al.*, 2022 ▸; Labib *et al.*, 2026*b* ▸).

More refined analyses based on modified Arrott plots, scaling relations and Kouvel–Fisher methods yield critical exponents in the ranges β = 0.39–0.54 and γ = 0.89–1.17 (summarized in Table 1 ▸). Using Widom’s identity (Widom, 1965 ▸), δ = 1 + γ/β, the resulting values of δ fall within the range 2.66–3.85 for all examined PAs and QCs. These values are significantly smaller than those expected for conventional 3D universality classes, such as δ = 4.80 for the 3D Heisenberg model, δ = 4.82 for the 3D Ising model (Arrott, 1957 ▸) and δ = 5.00 for the tricritical mean-field model (Banerjee, 1964 ▸). Instead, they are remarkably close to the value γ/β = 3.00 predicted by the Landau mean-field model (Arrott, 1957 ▸) (see Table 1 ▸). The near-mean-field critical exponents in QCs and PAs indicate that the effective magnetic interaction remains long-range near *T*_C_, consistent with the RKKY-mediated exchange mechanism discussed in Section 2.

Recently, with the discovery of bulk annealable ferro­magnetic iQCs encompassing both Heisenberg (Gd-based) and non-Heisenberg (Tb- and Dy-based) systems, more reliable critical analyses have become possible owing to the significantly improved structural quality and thermal stability of these materials (Tamura *et al.*, 2026 ▸). The resulting critical exponents reveal a clear distinction between the two classes of magnetic systems. The non-Heisenberg Tb- and Dy-based iQCs exhibit critical exponents close to the mean-field values, indicating that their magnetic transitions are governed by effectively long-range interactions. In contrast, the Heisenberg Gd-based iQC displays reduced β and enhanced γ values, leading to a significantly larger δ exponent and a clear deviation from mean-field behaviour. This difference can be attributed to the distinct nature of spin fluctuations in the two systems. In the Heisenberg Gd-based iQC, the isotropic nature of the Gd^3+^ moment (*L* = 0) allows fluctuations in all spin directions, resulting in stronger critical fluctuations near *T*_C_. By contrast, the strong CEF anisotropy of Tb^3+^ and Dy^3+^ restricts the accessible spin degrees of freedom and suppresses transverse spin fluctuations, thereby stabilizing mean-field-like critical behaviour. Consequently, the Gd-based iQC exhibits intermediate critical exponents lying between the mean-field and 3D Heisenberg limits, highlighting the important role of local spin symmetry in determining magnetic criticality on quasiperiodic lattices.

### 7.2. Monte Carlo insights and new universality

A recent Monte Carlo study (Watanabe *et al.*, 2025 ▸) has provided important theoretical insight into the critical behaviour of Heisenberg-type magnetism in Tsai-type iQCs. In this work, the classical Heisenberg model was implemented on the magnetic sublattice of a representative Cd_5.7_Yb iQC and the critical properties were analysed using finite-size scaling techniques. The simulations yielded critical exponents β = 0.508, γ = 1.361 and ν = 0.792, which satisfy both the hyper­scaling and Rushbrooke relations, confirming the second-order nature of the transition commonly observed in experiments (Table 1 ▸). Notably, this exponent set differs from those of conventional 3D Heisenberg magnets, indicating that the quasiperiodic structure gives rise to a distinct universality class.

A key outcome of the study is the identification of cooperative magnetic correlations among the eight classes of nonequivalent RE sites inherent to the Tsai-type quasi­periodic structure. Rather than ordering independently, the spins associated with these inequivalent sites develop simultaneously at the transition, producing a collective FM state. This cooperative behaviour provides a microscopic explanation for the mean-field-like features observed experimentally in several Tsai-type PAs and QCs (Table 1 ▸). The results suggest that the hierarchical connectivity of nonequivalent sites in a quasiperiodic structure can mediate effective long-range interactions, even in the absence of translational periodicity, thereby stabilizing a unique critical regime distinct from both periodic magnets and disordered systems.

## 8. Future directions: toward designable quasiperiodic magnetism

### 8.1. Thermodynamically stable magnetic QCs

To date, most FM or AFM QCs have been obtained through rapid-quenching or arc-melting techniques (Tamura *et al.*, 2021 ▸; Takeuchi *et al.*, 2023 ▸; Tamura *et al.*, 2025 ▸), resulting in metastable and structurally imperfect phases that readily transform into periodic PAs or other crystalline phases upon annealing. This intrinsic instability severely limits the ability to improve quasiperiodic order and hinders precise investigations of fundamental magnetic properties, including anisotropy effects and critical behaviour. The discovery of thermodynamically stable magnetic QCs, particularly in single-crystalline form, would therefore represent a major breakthrough, enabling high-precision studies of magnetic structures and phase transitions under well controlled conditions. Such an achievement is expected to mark a turning point in the exploration and design of magnetism in quasiperiodic materials, analogous to An-Pang Tsai’s well known discovery of the first stable QC in 1987 (Tsai *et al.*, 1987 ▸) that revolutionized QC research.

### 8.2. Control knobs: elemental substitution, doping, magnetic field, pressure

Recent theoretical studies (Watanabe, 2021*a* ▸; Sugimoto *et al.*, 2024 ▸; Miyazaki *et al.*, 2020 ▸) have provided a unified microscopic picture of magnetism in Tsai-type QCs by employing point-charge analysis considering the CEF potentials of the ligand atoms around magnetic moments or Monte Carlo simulations. These reports unambiguously predict the emergence of nontrivial, nearly degenerate and competing magnetic textures. In the CEF-based framework, the average valence electron number of the nonmagnetic ligand atoms is identified as a key parameter controlling the orientation of local anisotropy axes and the resultant non­trivial spin textures. The Monte Carlo studies emphasize the role of the Fermi wavevector *k*_F_ as a governing parameter that determines the nature of the RKKY-mediated interactions and the resulting magnetic ground states. Experimentally, both parameters can be effectively tuned through chemical engineering and elemental substitution, since the Fermi wavevector is closely related to the valence electron concentration *e*/*a*. These results therefore highlight the central role of *iso- and heterovalent elemental substitution* as a practical strategy for controlling magnetic ordering in Tsai-type QCs and their PAs.

**Figure 24:**
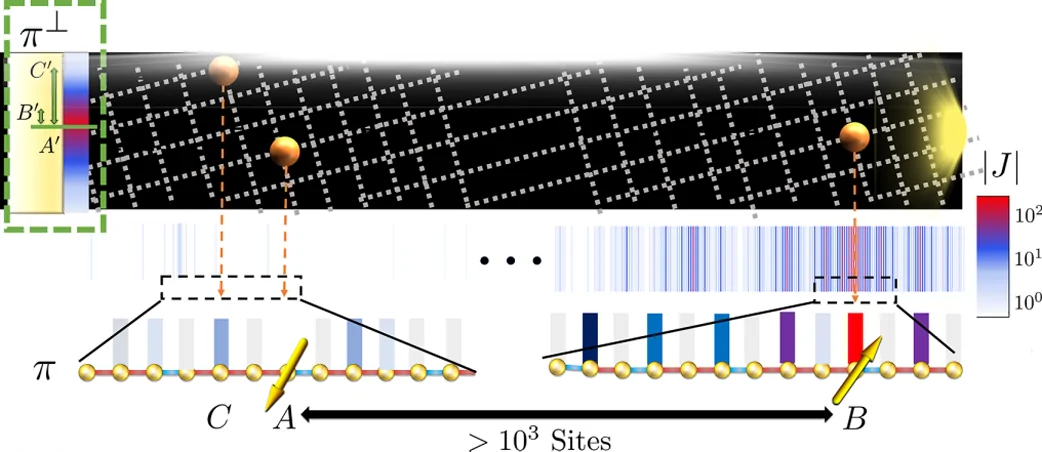
Schematic illustration and numerical calculation of long-range inter­actions in a quasiperiodic chain generated by a cut-and-project scheme. The quasiperiodicity originates from the irrational slope of the physical space (π) with respect to the 2D square hyperlattice (grey dashed lines). There is a one-to-one correspondence between the sites of the quasiperiodic chain and the hyperlattice sites, together with their projections onto the perpendicular space (π_⊥_), shown as the yellow bar. The quasiperiodic pattern consists of two (or more) nearest-neighbour hoppings, represented by red and blue bonds. Yellow spheres denote lattice sites. The white–blue–red peaks show the spatial distribution of the magnitude of the long-range interaction *J* for a magnetic impurity located on site *A* (yellow arrow). The plotted |*J*| values are relative magnitudes calculated for the silver mean tiling. Notably, the spin on site *B*, although separated from *A* by a large physical distance, couples more strongly to *A* than to a nearby site such as *C*. Such strongly coupled sites share similar local environments and therefore lie close to one another in perpendicular space, as indicated by the short π_⊥_ separation between A* and B*. The coupling strength decays primarily as a function of perpendicular-space distance, as emphasized by the green arrows. Reprinted with permission from Jeon & Lee (2025 ▸). Copyright (2025) by the American Physical Society.

A recent theoretical study has introduced a new perspective on long-range interactions in QCs by demonstrating that quasiperiodicity can fundamentally modify the spatial behaviour of indirect exchange couplings. Jeon & Lee (2025 ▸) still derived the effective spin–spin interaction within a conduction-electron-mediated (RKKY-like) framework but, unlike the simple analytical RKKY form for periodic Bloch systems, the interaction strength in quasiperiodic systems is strongly influenced by critical electronic states and hyperspace geometry. In particular, the study shows that the interaction does not necessarily decay monotonically with physical distance, but can instead be governed by a geometric metric defined in the perpendicular (hyperspace) dimension. As a result, two spins that are physically far apart can still exhibit strong coupling if their positions are close in the perpendicular space (see Fig. 24 ▸ for a schematic illustration). This mechanism enables anomalous enhancement of long-distance inter­actions.

From a materials design perspective, these results do not diminish the importance of electronic parameters such as *k*_F_ or *e*/*a*. Rather, since the exchange interaction remains mediated by electronic states near the Fermi level, tuning *e*/*a* can still strongly influence the sign, strength and competition of magnetic interactions through modifications of the underlying electronic structure. At the same time, the work suggests an additional degree of freedom unique to QCs, namely hyperspace geometry, for engineering long-range magnetic couplings and quantum functionalities. In other words, hyperspace geometry-induced long-distance quantum coupling makes it possible for long-range entangled states and unconventional collective phases to emerge by tuning the hyperspace distance by, for example, elemental doping. This opens opportunities for designing new QCs with targeted magnetic correlations, nonlocal spin control or quantum-coherent functionalities, and suggests that quasiperiodicity itself may serve as a design principle for realizing exotic phases such as quantum spin liquids, topological states or unconventional superconductivity.

**Figure 25:**
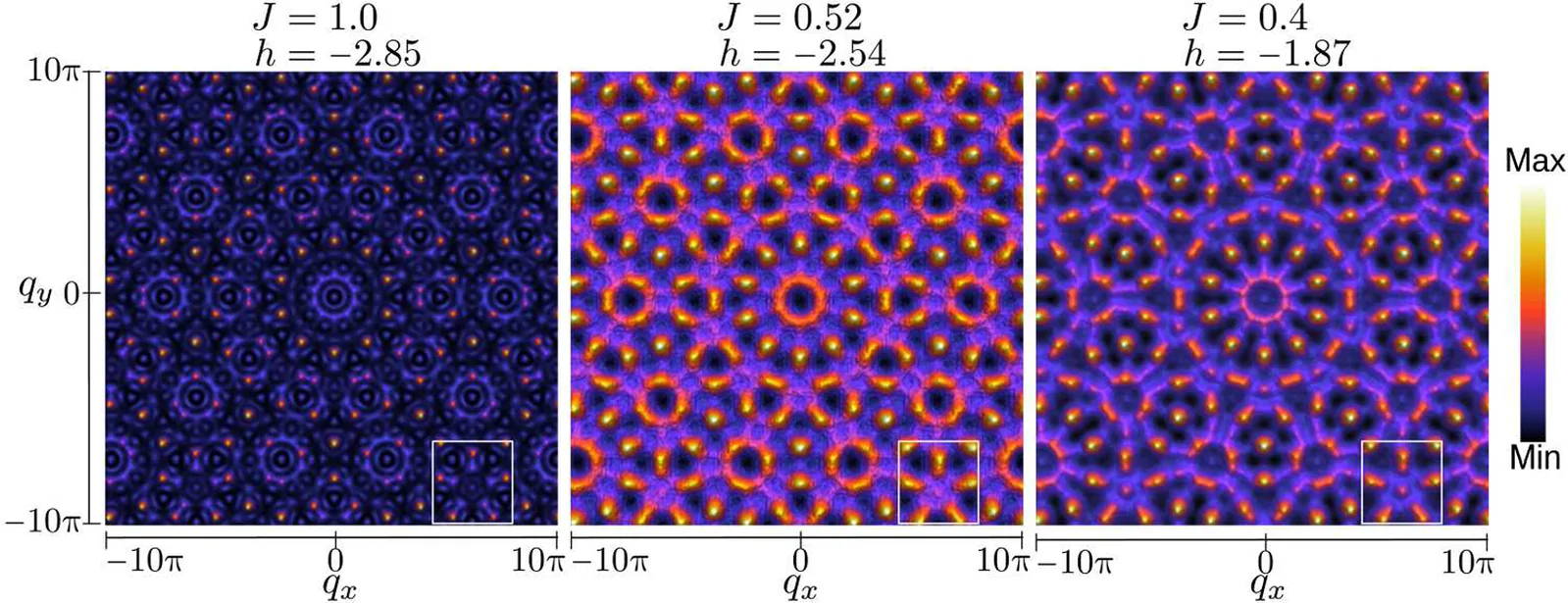
The rich variety of magnetic configurations is reflected in the structure-factor diagrams of various phases. White squares highlight some of the pentagonal shapes in reciprocal space denoting the fivefold symmetry of the QC. After Lopez-Bezanilla & Nisoli (2023 ▸).

A complementary design perspective has recently emerged (Lopez-Bezanilla & Nisoli, 2023 ▸), demonstrating that quasiperiodic structures can host a wide variety of magnetic phases controlled by external parameters, such as coupling strength and applied magnetic field. By implementing a Penrose QC on a programmable quantum annealer, the authors showed that the interplay between aperiodic connectivity, local inter­actions and external fields produces multiple frustrated magnetic configurations within a single finite structure. In this framework, different regions of the QC can dynamically activate or freeze their spins, leading to a sequence of field-induced magnetic textures, including FM and ferrimagnetic modulations (see Fig. 25 ▸ for a few examples of emergent quasiperiodic long-range magnetic orders). These results demonstrate that the intrinsic structural aperiodicity of QCs provides a flexible platform capable of hosting multiple competing magnetic states, suggesting that quasiperiodic geometry itself can be exploited as a design parameter for engineering novel magnetic phases.

From a materials design standpoint, this work highlights a practical route for controlling magnetic states in QCs through the combined tuning of interaction strength and external fields. In real materials, the effective coupling strength corresponds to parameters such as the RKKY interaction scale, magnetic anisotropy or exchange pathways, all of which can be modified by chemical substitution, electron concentration or pressure. Similarly, the external longitudinal field in the model corresponds to experimentally accessible magnetic fields or internal molecular fields. The results therefore suggest a concrete design strategy: by controlling the balance between interaction strength and external or internal fields in a quasiperiodic structure, one can selectively stabilize different magnetic textures within the same structural framework. This approach opens a pathway toward multifunctional magnetic QCs capable of hosting multiple stable or switchable magnetic states, with potential applications in information storage, spintronic devices and quantum information platforms.

### 8.3. Data-driven discovery and machine learning

The vast compositional and structural space of QCs presents a major challenge for conventional trial-and-error approaches, particularly when magnetic ground states arise from subtle competition among multiple interactions. In this context, machine-learning and data-driven methods have recently emerged as powerful tools for accelerating the discovery of QCs and related PAs. Composition-based machine-learning models have demonstrated the ability to predict QC-forming alloys with high accuracy, enabling efficient exploration of previously uncharted alloy systems beyond traditional empirical guidelines such as the *e*/*a* rule (Liu *et al.*, 2023 ▸; Liu *et al.*, 2021 ▸). In parallel, deep-learning techniques have been successfully applied to identify QC phases directly from complex powder diffraction data, significantly reducing experimental bottlenecks and enabling high-throughput validation of predicted candidates (Uryu *et al.*, 2024 ▸). These developments point towards a closed-loop data-driven framework in which machine-learning-assisted composition screening, automated phase identification and targeted synthesis are seamlessly integrated. Extending such approaches to incorporate magnetic descriptors, such as ordering temperatures, anisotropy scales and magnetic structure motifs, offers a promising route for discovering and designing magnetic QCs and PAs and for transforming QC magnetism research field into a predictive design-oriented discipline.

## 9. Conclusions

Aperiodic crystals provide a fundamentally distinct platform for magnetism because long-range structural order exists without translational periodicity. Among them, quasicrystals (QCs) have attracted significant attention since their discovery in 1982. Since then, numerous theoretical studies on quasiperiodic lattices and frustrated magnetic models have predicted rich magnetic phenomena, including noncoplanar magnetic structures, spin-ice-like states and multipolar magnetism. Importantly, these studies have consistently established that quasiperiodicity itself does not fundamentally prohibit long-range magnetic order. Nevertheless, for several decades after the discovery of QCs, all magnetic QCs exhibited only spin-glass-like freezing, raising the long-standing experimental question of whether long-range magnetic order could actually be realized in real quasiperiodic materials. This issue was only recently resolved through the experimental discovery of long-range ferromagnetic and antiferromagnetic orders in real icosahedral QCs.

This review provides three major design guidelines for understanding and controlling magnetism in Tsai-type QCs and their periodic approximants (PAs). The first is tuning the valence electron concentration (*e*/*a*) to control the sign and magnitude of RKKY-mediated exchange interactions. The second is exploiting crystal electric field (CEF) anisotropy to stabilize noncollinear magnetic structures. The third is controlling structural degrees of freedom associated with frustration, disorder and rare earth network connectivity. Many of these design principles have emerged primarily from detailed investigations of periodic approximants, and therefore PAs have been extensively discussed throughout this review, together with numerous case studies of magnetic order in both QCs and PAs.

The present review further discusses experimentally refined whirling magnetic orders in non-Heisenberg Tsai-type 1/1 PAs and the conditions required for their stabilization. Strong Ising-like single-ion anisotropy, primarily encoded by the local atomic environment, together with ferromagnetic intra-cluster interactions, are introduced as key parameters stabilizing these noncoplanar whirling magnetic structures. The review further summarizes recent progress in theoretical simulations and emergent magnetic textures predicted in QCs and PAs, including whirling, antiwhirling and hedgehog-like states. Point-charge analyses under CEF-induced anisotropy indicate that these noncoplanar magnetic textures originate primarily from the local icosahedral cluster geometry and competing magnetic interactions common to both PAs and QCs, while quasiperiodicity acts as an additional structural degree of freedom influencing the long-range stability of magnetic states.

Critical behaviour near magnetic phase transitions, recent theoretical developments, emerging opportunities and outstanding challenges, including the determination of microscopic magnetic structures in most magnetic QCs and the role of quasiperiodicity in magnetic excitations and critical phenomena, have also been discussed throughout this review. Overall, the results summarized here demonstrate that Tsai-type QCs and PAs provide a powerful platform for investigating emergent magnetism in structurally complex materials, and they open broad opportunities for discovering novel magnetic states in quasiperiodic systems.

## Figures and Tables

**Table 1:** Critical exponents β, γ and δ and Curie temperature *T*_C_ for various Tsai-type PAs and QCs and theoretical models

Composition	β	γ	δ	*T*_C_ (K)
Au_54_Cu_7.5_Al_12_In_12_Gd_14.5_ iQC (Tamura *et al.*, 2026 ▸)	0.41	1.17	3.85	26.9
Au_57.5_Cu_5.5_Al_10.5_In_12_Tb_14.5_ iQC (Tamura *et al.*, 2026 ▸)	0.51	0.91	2.78	16.5
Au_57.5_Cu_5_Al_13_In_10_Dy_14.5_ iQC (Tamura *et al.*, 2026 ▸)	0.47	0.89	2.89	9.6
Ga_40_Au_21_Pt_25_Gd_14_ 1/1 PA (Labib *et al.*, 2025 ▸)	0.50	0.97	2.94	8.8
Ga_30_Au_33_Pt_23_Gd_14_ 1/1 PA (Labib *et al.*, 2025 ▸)	0.45	1.01	3.24	12.8
Ga_31_Au_35_Pt_20_Gd_14_ 1/1 PA (Labib *et al.*, 2025 ▸)	0.44	0.99	3.25	14.9
Ga_28_Au_33_Pt_25_Gd_14_ 1/1 PA (Labib *et al.*, 2025 ▸)	0.43	1.03	3.39	8.8
Au_72.7_Si_13.6_Gd_13.7_ 1/1 PA (Shiino *et al.*, 2022 ▸)	0.47	1.12	3.38	21.8
Au_68.6_Si_16.0_Gd_15.4_ 1/1 PA (Shiino *et al.*, 2022 ▸)	0.51	1.00	2.96	16.8
Au_56.4_Al_10.8_Cu_6.9_In_11.8_Tb_14.1_ 1/1 PA (Labib *et al.*, 2024*b* ▸)	0.40	0.90	3.25	15.8
Au_56.1_Al_10.5_Cu_6.9_In_11.8_Tb_14.7_ 2/1 PA (Labib *et al.*, 2024*b* ▸)	0.39	1.08	3.77	14.3
Au_65_Ga_20_Dy_15_ iQC (Takeuchi *et al.*, 2023 ▸)	0.54	0.89	2.66	7.5
Au_60_Ga_26_Tb_14_ 1/1 PA (Labib *et al.*, 2024*a* ▸)	0.42	–	–	15.5
				
Icosahedron (Monte Carlo) (Watanabe *et al.*, 2025 ▸)	0.508	1.361	3.67	–
Tricritical mean-field	0.25	1.00	5.00	–
Mean field	0.50	1.00	3.00	–
3D Ising	0.325	1.24	4.82	–
3D Heisenberg	0.365	1.386	4.82	–
